# Cell-specific imputation of drug connectivity mapping with incomplete data

**DOI:** 10.1371/journal.pone.0278289

**Published:** 2023-02-16

**Authors:** Diana Sapashnik, Rebecca Newman, Christopher Michael Pietras, Di Zhou, Kapil Devkota, Fangfang Qu, Lior Kofman, Sean Boudreau, Inbar Fried, Donna K. Slonim

**Affiliations:** 1 Department of Computer Science, Tufts University, Medford, MA, United States of America; 2 Department of Medicine, University of North Carolina School of Medicine, Chapel Hill, NC, United States of America; 3 Department of Immunology, Tufts University School of Medicine, Boston, MA, United States of America; Bocconi University: Universita Bocconi, ITALY

## Abstract

Drug repositioning allows expedited discovery of new applications for existing compounds, but re-screening vast compound libraries is often prohibitively expensive. “Connectivity mapping” is a process that links drugs to diseases by identifying compounds whose impact on expression in a collection of cells reverses the disease’s impact on expression in disease-relevant tissues. The LINCS project has expanded the universe of compounds and cells for which data are available, but even with this effort, many clinically useful combinations are missing. To evaluate the possibility of repurposing drugs despite missing data, we compared collaborative filtering using either neighborhood-based or SVD imputation methods to two naive approaches via cross-validation. Methods were evaluated for their ability to predict drug connectivity despite missing data. Predictions improved when cell type was taken into account. Neighborhood collaborative filtering was the most successful method, with the best improvements in non-immortalized primary cells. We also explored which classes of compounds are most and least reliant on cell type for accurate imputation. We conclude that even for cells in which drug responses have not been fully characterized, it is possible to identify unassayed drugs that reverse in those cells the expression signatures observed in disease.

## 1 Introduction

*Connectivity mapping* [1] refers to the process of drug repositioning by finding candidate drugs that best reverse the expression changes caused by a given disease or condition. The original Connectivity Map database used microarrays to profile gene expression changes in up to four cancer cell lines treated with 164 perturbagens, many of them FDA-approved drugs. An expression “signature” from a given disease state, essentially sets of up- and down-regulated genes in relevant tissues from patients with the disease compared to normal controls, could then be used to find database compounds whose effect on gene expression was negatively correlated with the expression changes caused by the disease. Some compounds identified in this way were shown in further studies to have high potential for therapeutic efficacy in disease [1–3].

More recently, the LINCS Consortium dramatically scaled up the connectivity map database using the L1000 assay, which measures expression of 978 genes at a much lower cost. The LINCS connectivity map includes a much larger set of compounds, small molecules, and cellular perturbations across a wider range of cell types [4]. However, while there are now many more cells profiled in the connectivity database, the database is still very sparse, with most drug profiles in a small set of cancer cell lines. Yet recent work has shown that even different breast cancer cell lines can have different, context-specific responses to perturbation [5]. We observe that variation in primary cells’ responses is even greater.

Further, gene expression profiles in primary cells are a relatively small part of the LINCS data set, and profiling all desirable cell types and states is impractical even with more efficient assays. Thus, candidate drugs identified through connectivity mapping may have very different effects *in vivo*. For a precision medicine approach to connectivity, particularly outside the realm of oncology, the ability to identify drugs that reverse a patient’s disease signature even in the absence of connectivity data for a given cell/drug combination will improve scalability and relevance. We therefore aim to determine whether accurate imputation of context-specific connectivity is possible despite missing data.

The first step is imputing expression values for drug/cell combinations that lack experimental data. Since the early days of microarrays, there have been efforts to impute missing expression values caused by array defects or hybridization issues (e.g., array scratches, localized manufacturing defects, reagent spatters). The naïve approach, averaging over expression values for a given gene in other samples in the data set, was quickly improved upon by more principled methods, including k-nearest neighbors and SVD [6], local least squares optimization [7], and Bayesian prediction [8]. Several methods made use of time series information when available [6, 9, 10]. Collaborative filtering methods have even been applied to this problem [11, 12].

However, nearly all of these approaches address a different problem—one in which there is a limited fraction (e.g. under 20%) of missing genes for a given sample, and in which the missing data points are not correlated across samples. A few approaches try to fit some characteristics of the random missing data, such as the observed histogram of the fraction of missing genes per sample [8]. Few of the time series methods (e.g. [10]) deal with imputing whole expression profiles. Furthermore, to impute entirely missing expression profiles, one must incorporate additional domain information, such as data from nearby time points or functional relationships between genes’ expression patterns. In another example, transfer learning has been used to impute entire bulk RNA-sequencing profiles when methylation profiles for the same samples are available [13]. Here, we use expression profiles of related drugs and cells.

Explicitly imputing connectivity for unassayed drugs and cells is thus a distinct problem. Hodos, *et al*. [14] first described this problem and are the only others we know of to have addressed it. They performed distinct experiments from ours, assessing results both by expression imputation and by ability to predict drug targets or mechanism of action. In our work, which uses similar but independently-developed methods (differences are further addressed in the Discussion), we specifically evaluate the ability to connect drugs to query signatures in the absence of drug profiles, the amount of data required, and the drug classes that are more or less amenable to prediction with different methods. Our results therefore help define the experiments needed to better identify compounds that reverse cell- and condition-specific expression effects of medical conditions outside oncology.

## 2 Materials and methods

### 2.1 Overview of approach

To assess our ability to determine connectivity with missing data, we need a data set where we know the right answers. We create two such data sets by taking a complete subset of the LINCS connectivity data and a more realistic sparse subset in which approximately 75% of the drug/cell pairs are missing. Perturbations in LINCS include gene overexpression or inhibition, but for the purposes of this evaluation, we choose perturbations reflecting treatment with named compounds or chemicals. We evaluate performance on each data set through five-fold cross-validation to assess how well connectivity queries can produce the same results with missing data as they would with the full data set. Specifically, given a drug-cell combination of interest for which the expression profile is unavailable, we ask how well we can impute drug connectivity for this drug-cell pair, and whether we can do so more accurately by taking cell type into account.

### 2.2 Connectivity mapping data and queries

Let *C* be a set of *c* cell types, *D* be a collection of *d* drugs or cellular perturbations, and *N* be a set of *n* genes. We will refer to individual drugs or cells, such as the *i*th of |*D*| drugs or the *j*th of |*C*| cells, as *d*_*i*_ and *c*_*j*_, respectively. We start with a 3-dimensional matrix or tensor, *M*, containing gene expression profiles of all genes in *N* in a subset of the drug/cell combinations (*D* × *C*). These expression values reflect expression in the indicated cell treated with the indicated drug, compared to expression in the same cell without any treatment. Note that *M* may be sparse, but the expression profiles are all-or-nothing; if we have any expression data for a cell/drug combination, we have values for all *n* genes in the treated cell compared to the untreated cell.

As described in the paper introducing the initial L1000 connectivity data set [4], these expression changes are represented by z-scores. Specifically, we use the published “Level 5” data, which include z-scores of expression changes in drug-treated cells relative to controls, averaged over at least three replicates.

A *query signature* for a particular phenotypic state is defined to consist of two gene sets, one containing the most up-regulated, and the other the most down-regulated, genes in that state compared to a suitable control. So for example, a query signature for prostate cancer might consist of the 50 most up- and down-regulated genes in tumors from prostate cancer patients compared to adjacent normal tissue. Following the notation of [15], we use $S_{X}^{+}$ to refer to the set of the *k* most upregulated genes in the query comparison, and $S_{X}^{-}$ to refer to the *k* most downregulated genes for the same comparison.

A connectivity map query is performed in the following way, as described in more detail in [4]. Given the query signature $S_{X}=S_{X}^{+}\cup S_{X}^{-}$, and a reference expression profile $\vec{R}$ characterizing rank-ordered differentially-expressed genes in cell *c* treated with drug *d* compared to cell *c* without drug exposure, we compute the weighted connectivity score (WCS) as:

$$\text{WCS}=\{\begin{array}{cc} \frac{{\text{ES}}_{up}-{\text{ES}}_{down}}{2} & \text{if}\;\text{sign}\left({\text{ES}}_{up}\right)\neq \text{sign}\left({\text{ES}}_{down}\right)\\ 0 & \text{otherwise} \end{array}$$

where ES_*up*_ and ES_*down*_ refer to the weighted Kolmogorov-Smirnov enrichment statistic (ES) described in [16] that captures the enrichment of the set of genes *S*_*X*_ in reference profile $\vec{R}$. The WCS score ranges from -1 to 1. A score of 1 represents high positive connectivity, meaning that the drug’s effect on the given cell appears to be similar to that of the query signature, while a score of -1 represents high negative connectivity, or a drug/cell combination that up-regulates the down-regulated genes from the query signature and down-regulates its up-regulated genes.

To further enable comparisons across cells and drugs, we can compute the normalized connectivity scores (NCS) as in [4], by mean-scaling the WCS for a given cell *c* and drug *d*:

$$\text{NCS}=\{\begin{array}{cc} \text{WCS}/\mu_{c,d}^{+} & \text{if}\;\text{sign}(WCS)>0\\ \text{WCS}/\mu_{c,d}^{-} & \text{otherwise} \end{array}$$

where $\mu_{c,d}^{+},\mu_{c,d}^{-}$ are the absolute values of the means of the positive and negative WCS scores.

Our data are derived from the LINCS data published in the Gene Expression Omnibus (GEO) [17] as GSE70138 and GSE92742. Together these contain 591,699 expression profiles for 98 cell types and 29,668 perturbagens spread over 189,173 unique cell/perturbagen combinations.

For assessment purposes, we created two data sets: a small, complete data set, and a larger, sparse data set. The complete data set contains 12 cell types: 8 cancer cell lines, A375, A549, HCC515, HEPG2, HT29, MCF7, PC3, and VCAP; HA1E, an immortalized normal kidney cell line; one stem cell line, ASC (normal adipose-derived stem cells), and two types of primary neural cells: NPC (neural progenitor cells partially differentiated from iPSCs) and NEU (fully differentiated neurons). To create a complete and interpretable data set, we chose 450 drugs with “real” names (i.e., not just numbered compounds in development) and for which there is expression data for all 12 of the cell types above.

The sparse data set includes 80 cell types: 60 cancer cell lines, 6 immortalized normal cell lines, 4 stem cell lines, and 10 primary cell types. (S1 Table in S1 File lists the chosen cells and their classifications.) We selected a set of 1330 named drugs, again for interpretability, and included those drugs’ expression profiles for any of the 80 cells in which they were available. About 75% of the drug/cell combinations in this data set are missing, meaning that the compound was not assayed in that cell.

### 2.3 Data imputation methods

#### 2.3.1 Baseline methods

In the original connectivity map paper [1], connectivity scores were computed without consideration of cell type, essentially averaging across all cells. Given that only four cancer cell lines were used and that the goal of the project was to find common themes *across* the different cells, ignoring cellular context made sense. Even the current online connectivity tool, using the much larger and more varied LINCS data set, reports averaged “summary” profiles [4]. This standard informs the idea behind our baseline imputation methods.

**Fig 1:**
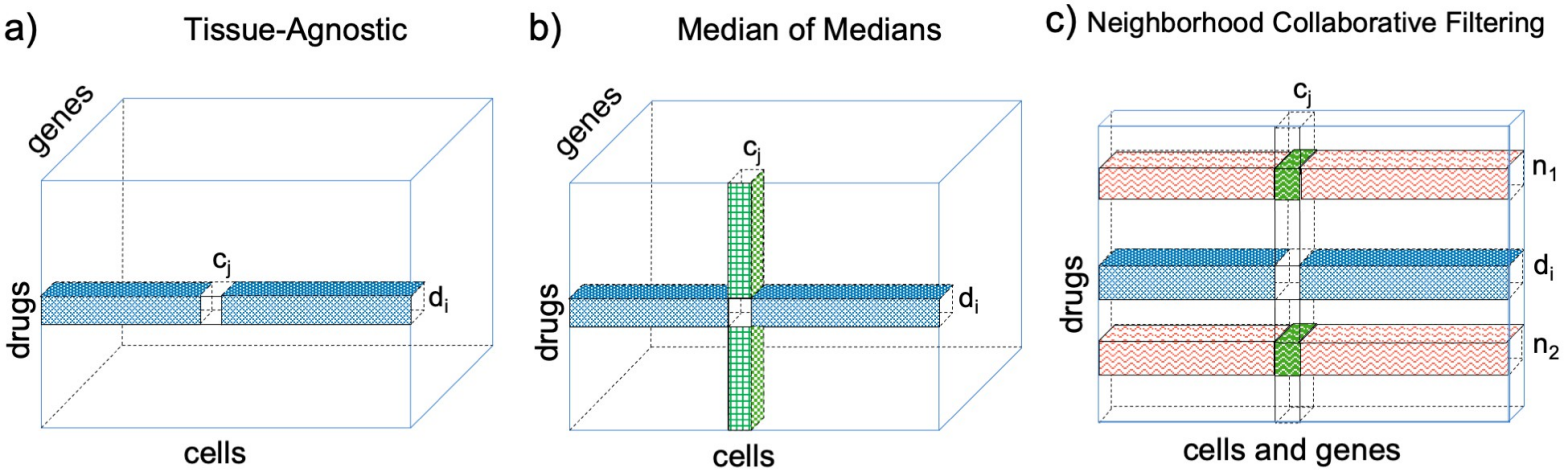
Methods overview. a) In Tissue-Agnostic imputation, the median of the expression of the first gene for drug *d*_*i*_ in cells other than *c*_*j*_ (the blue-colored row in the 3D matrix M) is used to predict the unknown expression profile for that cell/drug/gene combination (shown as a white missing value in the blue row). b) The Median of Medians (*MoM*) method predicts the same missing value by taking the median of two values: the median of that gene’s expression for drug *d*_*i*_ in cells other than *c*_*j*_ (the blue-colored row) and the median of that gene’s expression for drugs other than *d*_*i*_ in cell *c*_*j*_ (the green-checked column). c) In Neighborhood Collaborative Filtering, we use the flattened 2D matrix *R*, where rows contain values for all genes in cell 1, all genes in cell 2, and so on. Neighbors are drugs whose expression profiles on cells other than *c*_*j*_ (orange waves) are most similar to drug *d*_*i*_’s profile on cells other than *c*_*j*_ (the blue-colored row). In this example with *k* = 2 neighbors, the expression profiles for cell *c*_*j*_ on neighbor drugs *d*_*n*1_ and *d*_*n*2_ (darker / green waves) are used to impute the missing profile for drug *d*_*i*_ on cell *c*_*j*_.

**Tissue-agnostic (TA)**: A good baseline prediction of drug *d*_*i*_’s performance in cell *c*_*j*_ might be simply to look at what drug *d*_*i*_ does to a cell, regardless of what type of cell it is. Assume that we have expression profiles for drug *d*_*i*_ on other cell types. By taking the median of the expression values of that drug over all the other cells for which we do have data (the highlighted row in Fig 1a), we arrive at a prediction of what drug *d*_*i*_ “usually does” to a cell. We call this the *tissue-agnostic* imputation method, since it does not take the tissue or cell types involved into account. We compare our other results to this method, because it is similar to the tissue-agnostic average taken in the original Connectivity Map paper and the average across multiple cells reported in the “Summary” column by the LINCS connectivity tool at https://clue.io [4].

**Median of Medians (*MoM*)**. To include cell-type-specific information in a very straightforward way, we take the median of the tissue-agnostic prediction for drug *d*_*i*_ across all other cells with the analogous “drug-agnostic” prediction for *c*_*j*_ (the highlighted column in Fig 1b) that tells us how expression of *c*_*j*_ after perturbation typically differs from expression in other cells. We refer to this as the *MoM*, or Median of Medians, imputation method.

#### 2.3.2 Collaborative filtering

Collaborative filtering is an approach used in recommender systems to impute missing rating values and thereby recommend new products to users based both on information from similar users and from other items that user has rated. Calculations are typically based on sparse databases with *m* users and *n* items containing those users’ ratings for ≤ *n* of those items [18]. These ratings can represent any kind of relationship between users and items. In applications such as movie or purchase recommendations, the ratings might be represented by integers in the range [1, 5]. But in other applications, ratings might be real numbers or categorical variables. This approach is of particular interest for imputation of connectivity data because of the sparsity of the database. Our code uses the *rrecsys* package in R for this purpose.

**Neighborhood Collaborative Filtering (NCF)**: One approach to collaborative filtering is to rely on the closest neighbors of a particular sample as a model. An average, weighted by similarity of the neighbors’ ratings, approximates the rating for the sample of interest. A critical change was necessary to use this approach with our data, because each “rating” in the connectivity matrix is actually a vector of gene expression values. To avoid loss of information, we view the gene expression values as a multi-part rating of the same item. In the user/movie-rating metaphor, these values would represent a rating that perhaps specified an overall rating, as well as ratings of the movie’s acting, cinematography, and soundtrack.

Because these methods are intended for two-dimensional matrices, we create one from our three dimensional tensor *M* by concatenating, for each drug, expression values for all genes in the first cell, the second cell, etc. We call this reshaped matrix *R*. The *rrecsys* package then mean-centers each row and calculates similarities between drugs (rows) by taking the adjusted cosine similarity [19] between all pairs of rows.

For each missing expression profile, we then predict that profile by finding the top *x* “neighbors” of the row containing the missing value (Fig 1c). We used *k* = 50 neighbors for the complete 12x450 data set and *k* = 120 neighbors for the sparse 80x1330 data set; both are round numbers at around ten percent of the number of rows in the matrix. (We tuned the fraction-of-neighbors parameter once on a smaller and non-overlapping set of drugs; results were not highly sensitive to changes in *k*. Additionally, we replicated our results for the 80x1330 data set with *k* increased or decreased by a factor of two, and again found minimal effects on the connectivity results (S2 Fig in S1 File).) Prediction is then done using the weighted average of these predicted neighbors, weighted by their similarity scores, as in [19].

**Singular value decomposition (SVD)**: The goal of this approach is to account for latent subclasses within the drugs or cells. We used the *FunkSVD* R package, which applies stochastic gradient descent SVD optimization to build an approximation of an input matrix. This specific methodology was shown to be particularly effective in predicting Netflix movie ratings [20]. The FunkSVD method does not require a complete matrix to run, and effectively “overlooks” missing or unknown ratings [21]. Note that this is not the case for a simple SVD decomposition, which would require some initial “guess” for the missing entries [22]. Because FunkSVD expects a two-dimensional ratings matrix, the input is the reshaped matrix *R* defined above.

FunkSVD decomposes the matrix into component matrices *U* and *V* with singular values folded into those matrices. The parameters of this function determine the output rank of the approximation. For our purposes we used rank parameter *k* = 55. (As with the nearest neighbors approach, we tuned the rank parameter *k* once on an older data set using a non-overlapping set of drugs, and we then evaluated performance for the 80x1330 data set when doubling or halving *k*. Again, we found that those changes in *k* did not greatly impact the results (S2 Fig in S1 File).).

A lower-rank approximation can then be obtained by reconstructing the matrix with *M* = *UV*′. We then predicted the values of missing data per row with the approximation *r*_new_ = *uV*′ where *u* is a row in *M* containing an unknown multi-part rating.

### 2.4 Evaluation

#### 2.4.1 Cross-validation

To assess the correctness of connectivity using different imputation methods, we need to be able to compare to cases in which we have access to the missing data, so that we can consider the connectivity results on the actual data to be the “right answers.” For both the complete and sparse connectivity matrices, we therefore performed cross-validation, in the following way.

Each cell / drug combination was randomly and independently assigned to one of five folds. We then verified that the candidate set of fold assignments had no fold where more than 75 percent of the cells for a given drug, or 75 percent of the drugs for a given cell, were assigned to that fold, ensuring that any method would be able to produce an imputed expression profile for any missing cell/drug combination. If this requirement was violated, fold assignments were completely regenerated at random until the requirement was met.

For each fold, a given method is provided the z-score normalized gene expression profiles for *only* the cell / drug combinations not in the fold, and must impute the expression profiles for cell / drug combinations that are in the fold. For the sparse data set, folds are assigned only over existing profiles for evaluation purposes. That is, missing data is assigned a fold value of zero, and predictions for such values do not factor into our accuracy assessments. Over all five folds, a given method will produce a single imputed profile for each cell / drug combination. We then compare the imputed profile to the true profile for that cell / drug combination, using the various scoring metrics described below.

To assess variance due to the randomness in this cross-validation procedure, we created five independent instances (“runs”) of cross validation data sets for both the complete and sparse matrices. We summarize our results across those five runs. Both the data sets and the folds used for cross validation in each run are provided with the supporting data.

#### 2.4.2 Scoring connectivity prediction

Once we have a predicted expression profile for a withheld drug-cell combination, we must assess how accurate our prediction is through some comparison to the actual withheld expression profile. Since our aim is to infer connectivity for cell-drug pairs for which we lack data, an informative evaluation method would be to compare the connectivity results from the true data with those obtained using imputed data.

There are two parts of a list of drugs returned by a connectivity query that are of interest. Drugs with the most positive connectivity scores are drugs that replicate the query signature; these are sometimes used to identify similar drugs, or to find compounds that might cause a similar change to that of the query expression profile as an adverse event. Drugs with the most negative connectivity scores are those that reverse the observed query signature; these are candidate therapeutics for an observed disease signature. Accordingly, we want to assess how well the most-positive, or most-negative, connectivity results from the imputed data match those from the withheld true data.

To do this, we use the Weighted Spearman rank correlation (WSpe) measure, defined by [23]. We use a weight function defined as

w(r)=2ϕ(r|μ=0,σ=Nϵ),

where *r* is the result rank, *ϕ* is the normal distribution of the form *ϕ*(*x*|*μ*, *σ*), and *ϵ* is a parameter with value between 0 and 1 controlling the “aggressiveness” of the curve.

In this work, we chose *ϵ* = 0.01, which applies weights of significant magnitude to approximately the top 20 results, after which weights diminish towards zero. We tested the effect of doubling or halving *ϵ* but saw minimal changes in the results (S3 Fig in S1 File).

For an example of how scoring works, suppose that M includes data on the impact of the *VEGF* inhibitor bevacizumab on MCF7 cells. We create a query signature *S*_*X*_ containing the 50 most up- and 50 most down-regulated genes in MCF7 after exposure to bevacizumab. We then use that signature to query MCF7 cells in each of the five cross-validation folds for the complete data set; in each fold, connectivity scores are imputed for approximately 1/5 of the drugs (the randomly-chosen drugs that were withheld in that fold, as described in Section 2.4.1). Combining the imputed values over all folds yields a list of *all* the drugs ranked by their imputed WCS values in MCF7 cells. We then compare this imputed profile to the true profile using the weighted Spearman rank correlation metric defined above.

### 2.5 Detecting drugs with similar functions

#### 2.5.1 Do predictions connect to true signatures of similar drugs?

Beyond comparison to true profiles, we can evaluate our performance by assessing the connectivity of a drug’s expression profile to other drugs within the same perturbagen class. A perturbagen class (PCL) is a group of drugs that share a mechanism of action or target genes. PCLs are defined by published literature and further refined by connectivity analysis to yield 171 classes, as described in more detail in [4]. PCL membership in this manuscript was obtained through personal communication with the authors of [4].

For a given compound, a query signature reflecting expression changes caused by treatment of cells with that compound is expected to return strong positive connectivity between that drug and others in the same PCLs.

To evaluate an imputation method, we can construct a query from an imputed signature and determine if drugs in the same PCL set are over-represented towards the top of the returned list, expressing strong positive connectivity.

To do this, we used the imputed expression profile for each drug, *d*, to create a query signature with the top and bottom 50 genes (the number recommended by [4]). We then use that signature to query the remaining drugs in the true data set, returning a list of weighted enrichment scores for each drug, ordered from most to least positively connected to drug *d*.

We adapt gene set enrichment analysis tools [16, 24] to function as a “drug set enrichment analysis,” evaluating the distribution of drugs from a perturbagen class within a connectivity result. Ideally the drugs other than the one that produced the query signature should be among the most connected compounds.

For this analysis, we require a minimum of three drugs per PCL. After removing drug classes with no more than 2 drugs *in the chosen query matrix*, there are 132 drugs of the 450 in the complete data set that are in the remaining perturbagen classes. In the sparse data set, 334 of the 1330 drugs are in PCLs.

An enrichment score (ES) is calculated as described in Section 2.2, weighted by connectivity scores from the query results. In this context, an ES captures enrichment of a PCL set in the connectivity result of a drug that is a member of that set. The normalized connectivity score (NCS) is then calculated (also as described in Section 2.2) to account for the varying set sizes. Significance of enrichment is determined by comparing the observed NCS to the NCS of 1,000 random permutations of the connectivity result. The fraction of the absolute value of the permuted NCS greater than, and thus stronger, than the absolute value of the observed NCS yields the normalized p-value [25]. (For negative connectivity scores this becomes the fraction of random NCS less than the observed NCS.).

For each drug/cell combination the imputed signature is queried against the true matrix. The distribution of the normalized connectivity scores calculated for each drug, pcl, and cell combination is compared across methods, with the expectation that the NCS should be strongly positive if strong connectivity is correctly detected.

#### 2.5.2 Finding similar unassayed drugs

With known drug classes, we can also assess how well we impute connectivity query results *to* drug/cell combinations that were not experimentally evaluated. This allows us to compare efficacy of connectivity imputation across cells and drug classes. Missing data is predicted from the true expression profiles using neighborhood collaborative filtering, chosen because we found it to be the best approach overall. Experimentally determined signatures are replaced with the average of their imputed profiles from each of the five runs to generate a completely imputed matrix from the sparse data set. We also created another completely imputed matrix using the tissue-agnostic method for comparison. Each signature is queried against the complete matrix, with strong positive connectivity between drugs in the same PCL correctly identified if the NCS score is positive and significant (meaning the normalized p value is less than 0.05). We measure the percentage of drugs significantly enriched in their PCL for every PCL/cell combination to assess our ability to impute connectivity query results even in the absence of experimental data, as well as to estimate the amount of data needed.

## 3 Results

### 3.1 Using cell-specific data improves imputation

To assess accuracy, we performed connectivity queries with query signatures containing the most dysregulated genes for each drug in the treated cell compared to untreated controls. Specifically, the query signatures consist of the 50 most up- and down-regulated genes for a given drug. Positive connectivity scores identify the drugs that induce expression changes most similar to those of the query drug; negative connectivity scores identify drugs whose effects on a cell reverse the effect of the query drug.

**Fig 2:**
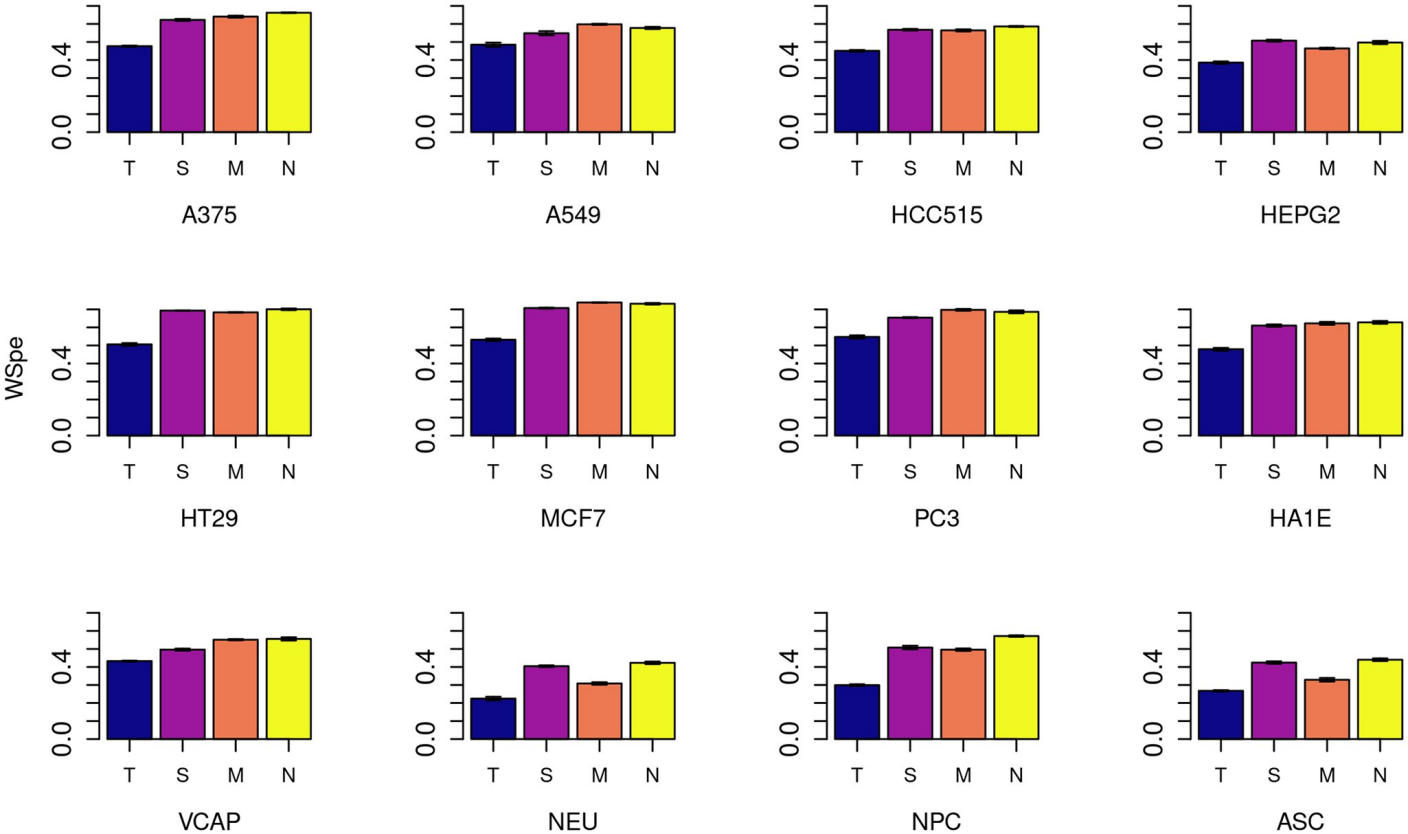
Negative connectivity results. Weighted Spearman rank correlation (WSpe) scores for negative connectivity across all genes and drugs, for each of the 12 cell lines in the complete data set. Methods are denoted by single-letter labels: T: Tissue-agnostic; S: SVD; M: Median of medians; N: Neighborhood collaborative filtering.

**Fig 3:**
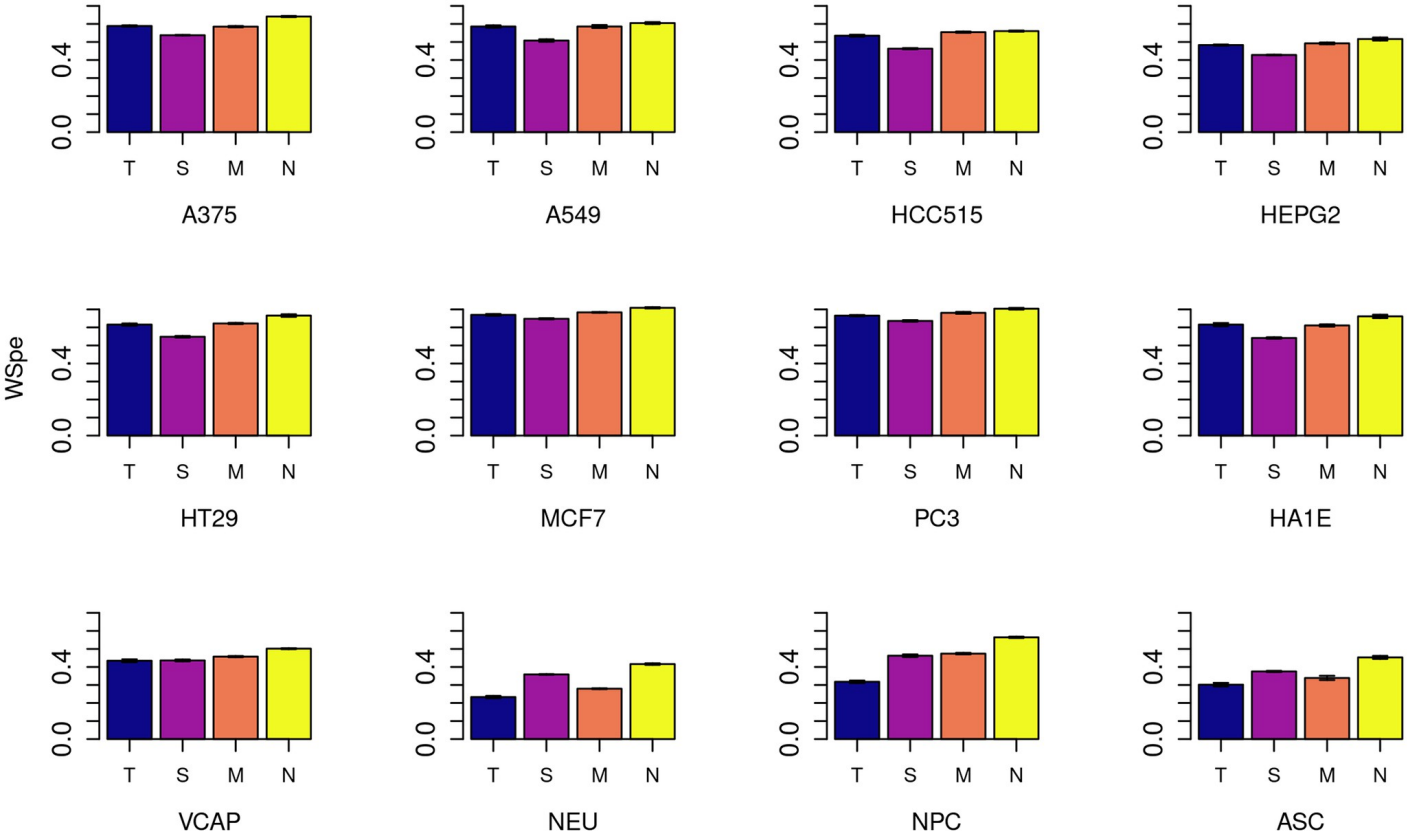
Positive connectivity results. Weighted Spearman rank correlation (WSpe) scores for positive connectivity across all genes and drugs, for each of the 12 cell lines in the complete data set. Methods are denoted by single-letter labels: T: Tissue-agnostic; S: SVD; M: Median of medians; N: Neighborhood collaborative filtering.

The average weighted Spearman rank correlations (WSpe) over all drugs between the true and predicted connectivity results for the complete data set appear in Fig 2 (negative connectivity) and Fig 3 (positive connectivity). S4, S5 Figs in S1 File show the percent changes in WSpe by cell, for each imputation method, compared to the tissue-agnostic method.

These figures show that for negative connectivity, the most commonly envisioned use case of the connectivity map, there is robust improvement in prediction under almost all methods, with neighborhood collaborative filtering either best or essentially tied with MoM for best in all cells. Neighborhood collaborative filtering’s improvements are especially large (> 80%, S2 Fig in S1 File) for the primary cell types NPC and NEU, where it notably out-performs MoM. But even in the cancer cell lines, an improvement of 20–40% over the tissue-agnostic baseline is seen with neighborhood collaborative filtering (Fig 2 and S4 Fig in S1 File).

Positive connectivity results show only a modest improvement over the tissue-agnostic baseline for all but the primary and stem cell types in the complete matrix (Fig 3 and S5 Fig in S1 File). Indeed, SVD is actually sometimes worse than tissue-agnostic imputation here. But the neighborhood collaborative filtering and MoM approaches, both of which use cell-specific information, are at least as good as the tissue-agnostic approach for all the cancer cell lines. For the primary and stem cells, neighborhood collaborative filtering still shows a substantial (> 50%) improvement, with MoM and SVD vying for second place. Note that the tissue-agnostic WSpe for NEU cells is the lowest (0.24) in this data set, with the other primary cell, NPC, in the low 0.30s, and most cancer cell lines having a WSpe between 0.5 and 0.67. Thus, obtaining a larger *percent improvement* over these much higher scores may be inherently harder.

Corresponding bar graphs for the sparse matrix results appear as S6-S9 Figs in S1 File. In these figures, cells are ordered both by cell type and, within each cell type, by the percentage of drugs that have been profiled experimentally for that cell. Consistent with the results from the complete data set, in the sparse data set there is improvement over tissue-agnostic for both positive and negative connectivity correlations across all classes of cells. Neighborhood collaborative filtering continues to outperform all methods for cells with enough data, with SVD and MoM improving over tissue-agnostic as well.

However, this trend begins to break down for cells where the percentage of drugs assayed is low. For primary cells, tissue-specific methods are more powerful in all cells with at least 41% of the 1330 drugs assayed. In this subset of the LINCS data, there are no primary cells with between 41% and 5% of the drugs assayed. In cells containing data for under 5% of the compounds, i.e., where there is not much cell-specific data to work with, the tissue-agnostic method is sometimes a more reliable approach, and error bars reflecting the varying cross validation folds become notably larger.

This holds true for cancer cell lines as well, where tissue-specific methods and particularly neighborhood collaborative filtering outperform tissue-agnostic imputation for cells with at least 48% of the compounds assayed, but become much less robust for cells in which fewer than 13% of the compounds have data, and here again there are large error bars. Immortalized and stem cells, though there are far fewer of them with reasonable amounts of data, appear to follow a similar pattern.

Together these results demonstrate that cell type is an important aspect of connectivity mapping. We thus generated boxplots (S10-S12 Figs in S1 File) summarizing the data from the bargraphs in Figs 2 and 3 (for the complete data set), and S6-S9 Figs in S1 File (for the sparse data set) by cell type. S10 Fig in S1 File shows this breakdown for the complete data set, confirming that the relative improvement is particularly large in primary cells. In S11 Fig in S1 File, we show the same plots for all cells in the sparse data set with data for at least 10% of the drugs, and we observe the same patterns. (We chose this cutoff because of the conclusions from Section 3.2.) For completeness, in S12 Fig in S1 File, we show the plot for all cells in the sparse data set regardless of the amount of data available. The patterns described above are still largely visible, but here they are partially obscured because the stem cell, primary cell, and immortalized cell categories are 50%, 60%, and 83% (respectively) comprised of cells with data for fewer than 10% of the drugs.

Overall, we conclude that incorporating cell-specific information improves connectivity outcomes in all cells, but it does so most dramatically in cells that are neither malignant nor immortalized.

### 3.2 Downsampling reveals amount of data needed

To determine how performance varies as a function of the number of compounds assayed, filling the gap between 41% and 13% identified in the previous section, we selected a few cells from each category of cells with more than 75% of the compounds assayed and downsampled the number of drugs. Compounds were randomly removed from folds until just 50%, 40%, 30%, 20% or 10% of the drugs remained for a given cell line. Signatures were imputed once more and negative connectivity compared to the true results. This was repeated across all five cross validation data sets.

**Fig 4:**
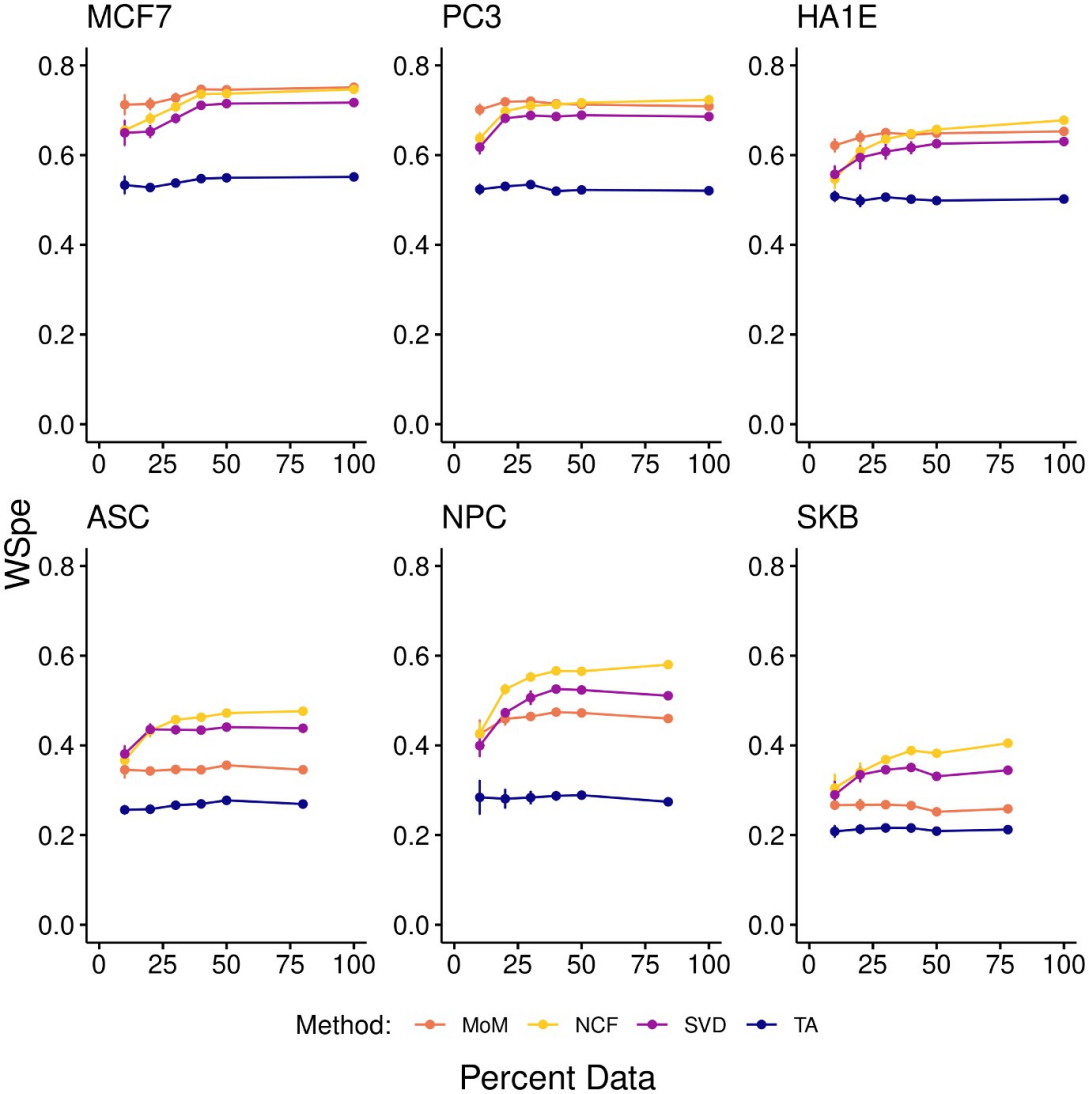
Downsampling. Negative WSpe for the named cells across all genes and drugs (y-axis), as a function of the percentage of the 1330 compounds whose data was available for imputation. Maximum x-values on each plot reflect the actual amount of data for that cell; all other points reflect downsampling. Error bars show the variation across cross-validation runs; most are smaller than the data markers.

Fig 4 shows the WSpe of true to imputed negative connectivity scores for each cell type averaged across all genes, drugs, and runs, plotted against the percent of compounds used for imputation. We found that in all cells analyzed, performance didn’t suffer notably so long as at least 20% of the drugs had data (e.g. we assayed approximately 260 of the 1330 compounds). Additionally, so long as data were available for at least 10% of the drugs, all other methods performed better than tissue-agnostic regardless of cell type, with Neighborhood collaborative filtering having the best performance or, in a few cases, being a close second.

These early findings suggest that for the larger, sparser data set, incorporating cellular context improves positive and negative connection detection over all cells types that have data for at least 130 drugs. Further work will be needed to determine more precisely how the distribution of compounds sampled affects predictive performance.

### 3.3 Queries from imputed profiles detect related compounds

**Fig 5:**
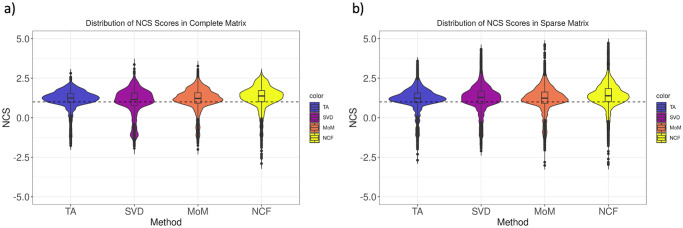
Distribution of connectivity scores for related drugs with imputed data. a) Violin plot showing distribution of normalized connectivity scores of drugs from a given drug set imputed from the complete matrix. b) Violin plot showing distribution of normalized connectivity scores of drugs from a given drug set imputed from the sparse matrix. Dashed lines show an NCS of 1.0. Abbreviations: NCS: Normalized Connectivity Score for drug-set enrichment analysis as defined in section 2.5.1; TA: Tissue-Agnostic; NCF: Neighborhood Collaborative Filtering.

Fig 5 shows the distribution of Normalized Connectivity Score (NCS) values between a drug and its corresponding Perturbagen Class (PCL) in all cells for the complete matrix (a) and sparse matrix (b), using the “drug set enrichment analysis” method described in section 2.5.1. In this context, a positive NCS means the connectivity query for drug *d* has drugs within the same PCL set clustered towards the top of the returned list, thus strong connectivity is accurately detected. For randomized lists, the NCS has an expected value of 0; if the imputed profiles successfully replicate the signature of drugs in the same PCLs, then we would expect the greatest density of NCS to be well above zero.

For all methods, in both the complete and sparse matrix, we find that the median of the NCS distribution is greater than 1.0 (represented in the plots by a dashed horizontal line), with the majority of NCS values clustered well above zero. Neighborhood collaborative filtering has a slightly higher median than all other methods and greater NCS values overall, proving to be the most robust and consistent method to be evaluated.

### 3.4 Predicting connectivity of unassayed drugs

We further generated a complete version of the sparse matrix (where 75% of the cell/drug combinations were missing) using only imputed data, as described in Section 2.5.2. We then assessed our ability to find related drugs within the same PCL using imputed query profiles against this completely imputed matrix.

**Fig 6:**
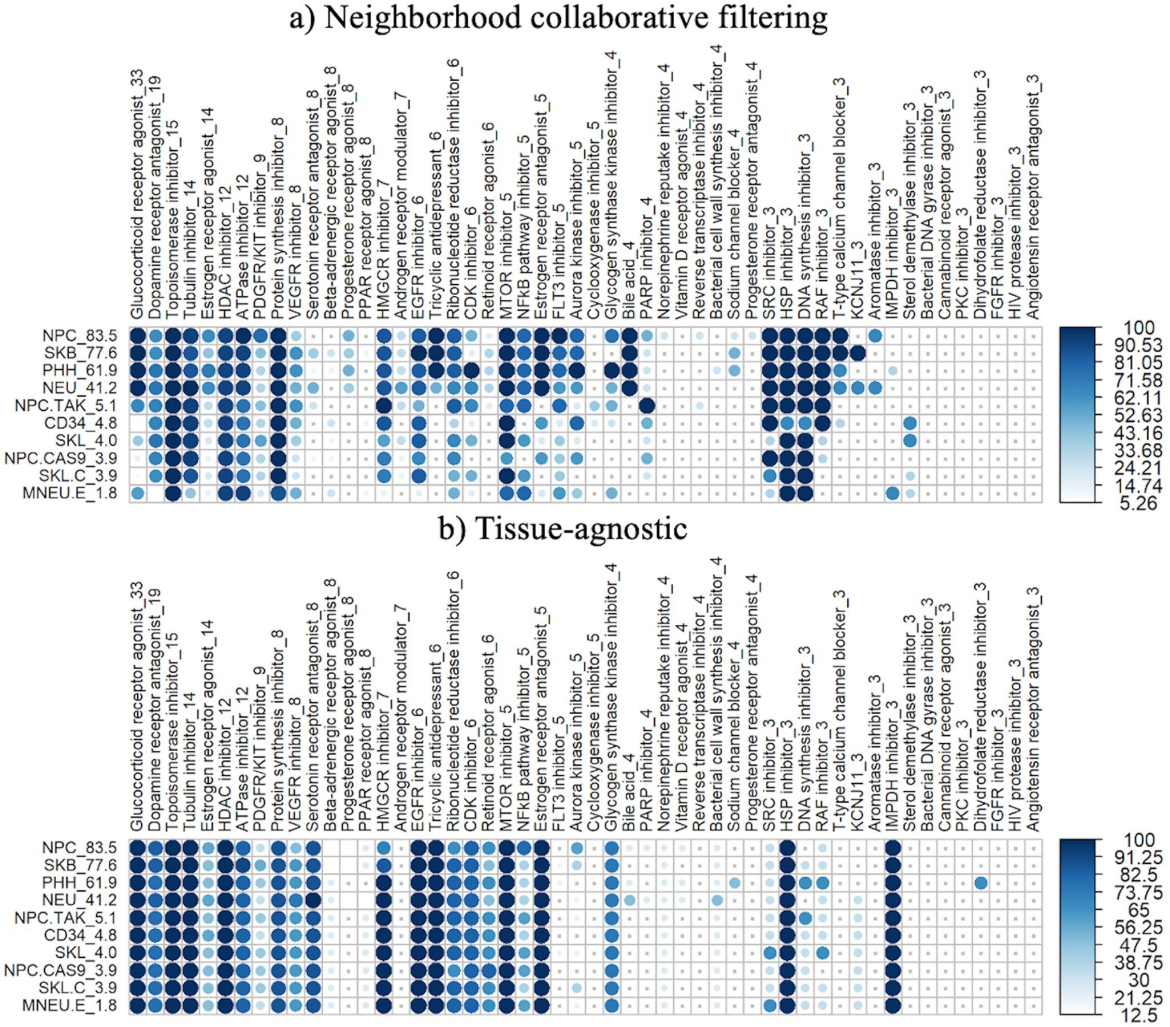
Recovery of imputed related drugs, by drug class and cell. a) Percent of drugs correctly expressing strong connectivity to their drug class using the fully imputed sparse matrix by the neighborhood approach, for primary cells only. The number of drugs in each PCL set is included in the PCL name, and the percentage of drugs assayed in each cell type is included in the cell name. Darker and larger dots correspond to a the higher percentage of drugs with statistically significant NCS values (Section 2.5.1). Tiny grey dots represent PCL/cell combinations in which there were no statistically significant NCS values. Plots were generated using the corrplot package [26]. b) Percent of drugs correctly expressing strong connectivity to their drug class using the fully imputed sparse matrix by the tissue-agnostic approach, for primary cells.

Fig 6a shows the percentage of drugs enriched for their PCL set using this complete matrix. Just primary cells are shown here; the full plots containing all 80 cells appear in S13, S14 Figs in S1 File. The full plots are organized by cell type (cancer, immortalized, stem and primary) and ordered by percentage of drugs profiled in each cell and by PCL size.

Of the drug/cell/PCL combinations, 53% have “drug set enrichment analysis” p-values (as defined in Section 2.5.1) less than 0.05, meaning that in the majority of drug and cell combinations evaluated, connectivity query results are correctly imputed by the neighborhood collaborative filtering method, given a sparse matrix of experimental data. The approach was best at accurately detecting strong positive connectivity in most of the drug sets in NPC, SKB, PHH and NEU cells, all of which had data for at least 41% of the compounds. In cells containing data for 5% or less of the compounds, the percentage of significant NCS values drops, consistent with the results noted above for positive and negative weighted connectivity correlations. This is true across all cell types assessed, with cells containing less than 5% of the 1330 drugs assayed showing a lower percentage of statistically significant NCS values.

Fig 6b shows the same plot for data imputed by the tissue-agnostic method, allowing us to identify classes of drugs in which imputation is better using cell-type-specific or tissue-agnostic methods. We notice that for tissue-agnostic imputation, the amount of data for the individual cell predictably doesn’t have much affect on accuracy; the bottom cells in the plot (with much less data) look more or less the same as those on top.

Similarly, drug classes with the most drugs tend be well imputed across the board, suggesting the likelihood that in such cases the “neighborhood” is well populated by related drugs. Drug classes for which imputation is better with neighborhood collaborative filtering include aurora kinase inhibitors, FLT3 inhibitors, and a number of steroid/hormone classes (progesterone receptor agonists, androgen receptor modulators). Classes where tissue-agnostic methods were better included retinoid receptor agonists and serotonin receptor antagonists. (These trends are visible in the primary cells in Fig 6 but may be clearer in S13, S14 Figs in S1 File, which show all the cells.).

We conclude that positive connections between drugs of the same class can be indeed be imputed for missing data. To do this well, more than 5% of the compounds from the 1330 assessed need to be experimentally evaluated for robust imputation, but again further work will be needed to determine more precisely the impact the number and distribution of compounds assayed has on accurate connectivity prediction.

### 3.5 Examples of predicted connectivity in disease

To illustrate use cases of our collaborative-filtering predictions, we identified differentially expressed genes in a previously-published microarray study that compared the bulk transcriptomes of postmortem hippocampus samples between subjects with schizophrenia and age- and sex-matched controls ([27]; GSE53987). We applied the fifty most up- and down-regulated genes as the “query signature” against a hybrid version of the sparse matrix, in which missing data was replaced by our cell-specific predictions. Our goal was to query both assayed and imputed data to identify compounds that might reverse this neuronal transcriptional signature despite not having been assayed in neurons (denoted NEU) in the LINCS data set.

Most of the top compounds reversing the schizophrenia signature had indeed already been directly profiled in neurons, so validating their links to schizophrenia simply supports the idea of connectivity mapping in non-cancer cells. Still, as an illustration, the top two such compounds were pirfenidone, an anti-fibrotic that has shown neurological effects in multiple sclerosis and chronic pain [28, 29], and remoxipride, an atypical antipsychotic indicated for schizophrenia, but whose use since 2003 has been limited due to toxicity concerns [30].

The most connected compounds *missing* from the sparse matrix, and that thus would not have been discovered without our predictive approach, include theophylline and bosentan. Theophylline is a naturally-occurring phosphodiesterase- and HDAC-inhibitor typically used in asthma but known to have neurological effects. It has been suggested for potential therapeutic use in both schizophrenia [31] and Alzheimer’s disease [32]. Bosentan is an endothelin receptor antantagonist whose targets, EDNRA and EDNRB, regulate migration of neural progenitor cells [33]; mouse mutations of these genes lead to abnormal neurological morphology and function (www.informatics.jax.org). These surprising yet plausible connections, along with more expected ones (e.g., citalopram, a selective serotonin reuptake inhibitor inexplicably not profiled in neurons), were discoverable only through predictive methods.

We next queried the same database with the signature of differentially expressed genes from a microarray study that compared left ventricular myocardial samples from ischemic cardiomyopathy patients to controls (GSE16499, [34]). There are no myocardial (heart muscle) cells in the LINCS data sets we downloaded, but there are primary skeletal muscle cells (SKL), although with data for only 4% of compounds their predictive power may be limited. Nonetheless, the most connected predicted compounds for the cardiomyopathy signature are givinostat, shown to have therapeutic efficacy in a mouse model of heart failure [35], and the ATP-ase inhibitor brefeldin-a, which affects cholesterol transport [36], a process important in cardiovascular disease. The one compound more significantly connected in muscle cells and actually assayed in the SKL cells was vorinostat (also known as suberoylanilide hydroxamic acid or SAHA), which has also shown benefit in a number of models of cardiac dysfunction [37].

## 4 Discussion

We have demonstrated that context-specific connectivity data can be used to infer missing connectivity data and can be reliably used to connect query signatures to drugs that may affect them even when those drugs have not been assayed in the relevant cells. This has applications for the full LINCS data set, in which many cell types have data for just a few tens of compounds, and even beyond. We have also shown that using cell-specific information does not always improve imputation of connectivity when under 5% of the drugs have been profiled in the cell—or in other words, when there isn’t much data to work from. An important direction for future work is to better understand the impact of the distribution of compounds needed for robust prediction across sparse matrices of different sizes and compositions.

After completion of this work, we became aware of a paper by Hodos, *et al*. addressing the same problem [14]. There are some differences in both the methods and the data sets we describe: their work starts with the level 3 LINCS data instead of level 5 [4], uses different evaluation metrics, and has a greater focus on positive connectivity and its utility for tasks such as predicting mechanism of action, whereas we explicitly focus on negative connectivity and its utility for identifying cell specific candidate therapeutics. Overall, the imputation methods used are quite similar, except that instead of using two-dimensional SVD to project the three-dimensional data set into a reduced space, Hodos *et al*. used a two-dimensional tensor completion method. They combined data from all possible ways of mapping the three dimensions into two, but then heavily downweighted one of them, essentially averaging the others.

Despite these differences, we see our work as a valuable complement to their study. We verified that cell type crucially influences connectivity results, and we found that having experimental data for at least 10% of the drugs appears to be enough for our cell-specific methods to out-perform the naive tissue-agnostic approach. We also confirmed that there is no one imputation method that performed best in all cases; performance relies on the specific cells involved.

An important novel contribution of ours is viewing the problem via the collaborative filtering framework, which we expect will suggest a host of additional methods to be tried in future work. Another difference appears in our interpretations of which perturbagen classes are more amenable to cell-specific or cell-agnostic imputation methods. We saw particular success with classes of kinase inhibitors and hormonal regulators. We also uniquely identified improved tissue-specific imputation in primary cells, demonstrating that using cell-specific methods and data will be especially important for extending connectivity methods beyond oncology. We expect that a detailed examination of specific results from both studies will provide additional information on best practices for future connectivity analyses.

Despite the successes of both studies, there is considerable room for improvement using new imputation methods. We found that the neighborhood collaborative filtering approach was most often the best method in this study. That said, there is the possibility of improving on SVD by using higher-order methods that better capture the three-dimensional structure of the data. The tensor formulation of Hodos, *et al*. is a good attempt in this direction, but current tensor completion methods do not generally handle missing data in *all* of one of the dimensions of the tensor (such as the absence of expression measurements of all genes for a given cell/drug combination). New approaches, including additional machine learning methods, might be more successful.

We also showed examples where queries based on actual expression data from patients were able to identify compounds that were not tested in those cells and that would not have been discovered without these predictive approaches. Of the four compounds highlighted as new discoveries in our example disease queries, only givinostat would have been among the top 10% of compounds if querying the “summary” profile, an average of just the assayed cells that is typically used when querying through the online tools provided by LINCS. The other compounds we highlighted would only have been identifiable through cell-specific imputation.

Finally, we conclusively emphasize that considering cell type is critical for accurate connectivity mapping. This point is also apparent in prior work with multiple different breast cancer cell lines [5], but it becomes even more important across more varied contexts. Both for precision cancer medicine purposes and for use in a wider range of medical conditions, taking context into account is therefore essential.

## Supporting information

S1 File This file contains all the supporting tables and figures. (PDF)
