# Supplementary material for: Cell-specific imputation of drug connectivity mapping with incomplete data
Source: PLoS One. 2023 Feb 16;18(2):e0278289. doi: 10.1371/journal.pone.0278289 (PMC9934325; doi:10.1371/journal.pone.0278289)
Supplement: S1 File — (PDF) [file pone.0278289.s001.pdf]

Supplement to "Cell-specific imputation of drug connectivity mapping with incomplete data"

D. Sapashnik, R. Newman, C.M. Pietras, D. Zhou, K. Devkota, F. Qu, L. Kofman, S. Boudreau, I. Fried, D.K. Slonim

August 22, 2022

Contents:

1. All 80 cells in the sparse data set organized by cell type (Table 1).
2. Comparison between halving and doubling parameter k: negative and positive connectivity (Fig. S2).
3. Comparison between various ϵ settings for the weighted correlation score between connectivity queries (Fig. S3).
4. Percent change in negative connectivity results compared to tissue-agnostic method: complete 12x450 data set (Fig. S4).
5. Percent change in positive connectivity results compared to tissue-agnostic method: complete 12x450 data set (Fig. S5).
6. Negative connectivity results for sparse 80x1330 matrix (Fig. S6).
7. Percent change in negative connectivity results compared to tissue agnostics: sparse 80x1330 matrix (Fig. S7).
8. Positive connectivity results for sparse 80x1330 matrix (Fig. S8).
9. Percent change in positive connectivity results compared to tissue agnostics: sparse 80x1330 matrix (Fig. S9).
10. Boxplots summarizing connectivity results by cell type: complete 12x450 data set (Fig. S10).
11. Boxplots summarizing connectivity results by cell type: sparse 80x1330 data set for all cells, including those with under 10% of the data (Fig. S11).
12. Boxplots summarizing connectivity results by cell type: sparse 80x1330 data set for all cells regardless of the number of drugs profiled (Fig. S12).

13. Percent of drugs enriched for PCL set in full neighborhood matrix: all cell types (Fig. S13).

14. Percent of drugs enriched for PCL set in full tissue agnostic matrix: all cell types (Fig. S14).

**Cancer**	**Primary**
MCF7, HT29, A375, PC3, A549, HCC515 ,	NPC
VCAP, HEPG2, HELA, YAPC, U937, LOVO,	SKB
SNUC4, SKMEL1, RMUGS, HCC15, HEC108, CORL23,	PHH
A673, NCIH596, TYKNU, SW948, SW620, SNU1040,	NEU
SNGM, SKMEL28, OV7, RKO, NCIH508, H1299,	NPC.TAK
AGS, EFO27, SW480, HCT116, JHUEM2, MDST8,	CD34
NCIH2073, COV644, DV90, RMGI, SKLU1, HT115,	SKL
WSUDLCL2, PL21, NCIH1836, NCIH1694, SNUC5, CL34,	NPC.CAS9
THP1, SKM1, T3M10, NOMO1, BT20, HS578T,	SKL.C
MDAMB231, SKBR3, HUH7, JURKAT, LNCAP, HL60	MNEU.E
**Immortalized**	**Stem Cell**
HA1E	ASC
HME1	FIBRNPC
MCF10A	ASC.C
HUVEC	HUES3
NKDBA	
HEK293T	

Table 1: Cells Types and Cells in Sparse Data Set.

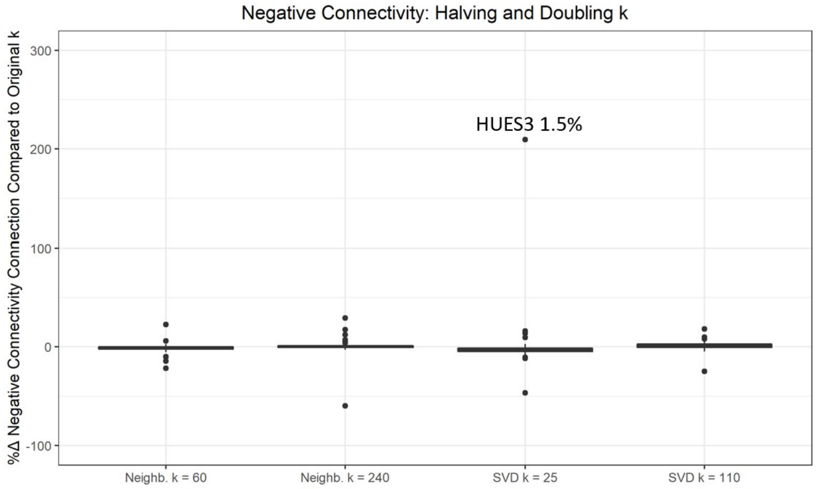

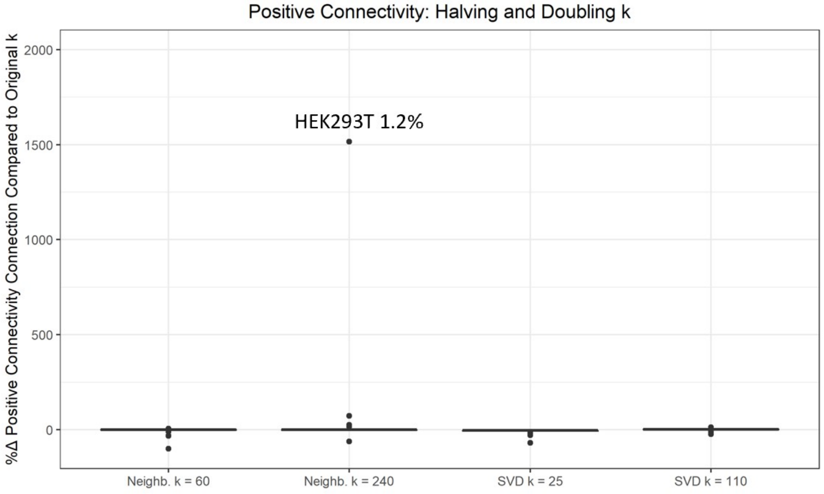

Figure S2: Percent change in WSpe for negative (top) and positive (bottom) connectivity across all drugs and genes obtained by varying k by a factor of two from the values of $k = 120$ for nearest neighbors and $k = 55$ for svd used in this work for the sparse data set. Outlier cell lines labelled with cell name and % data available for that cell.

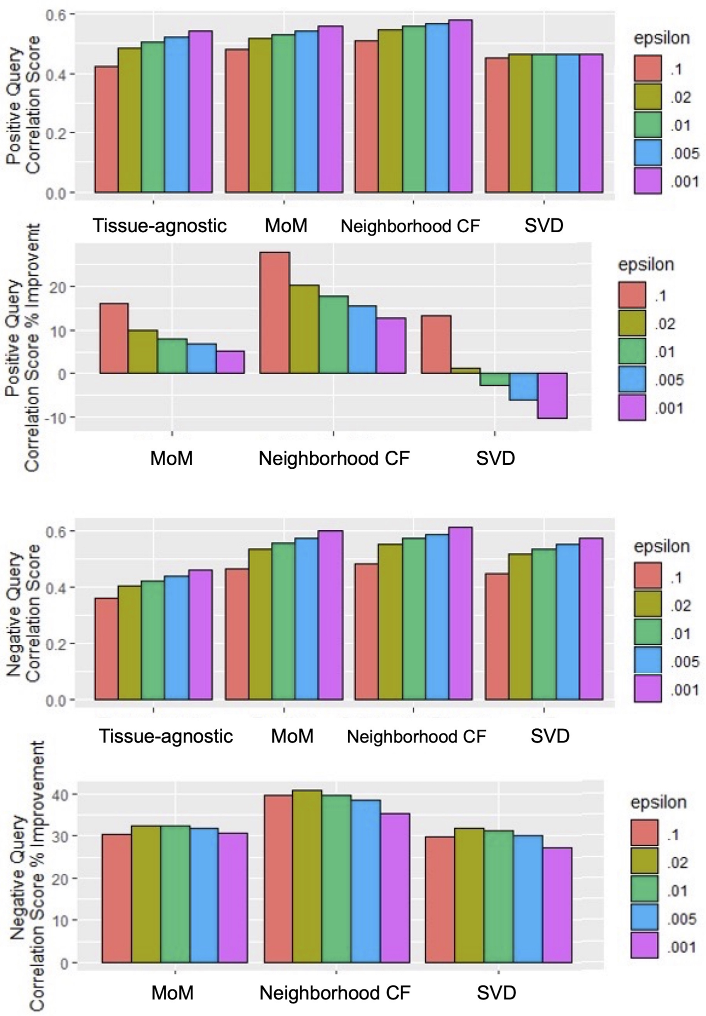

Figure S3: Change in average positive and negative query correlation scores across all cells and drugs obtained by varying ϵ by a factor of either two or ten in either direction from the values of $\epsilon = .01$ used in this work.

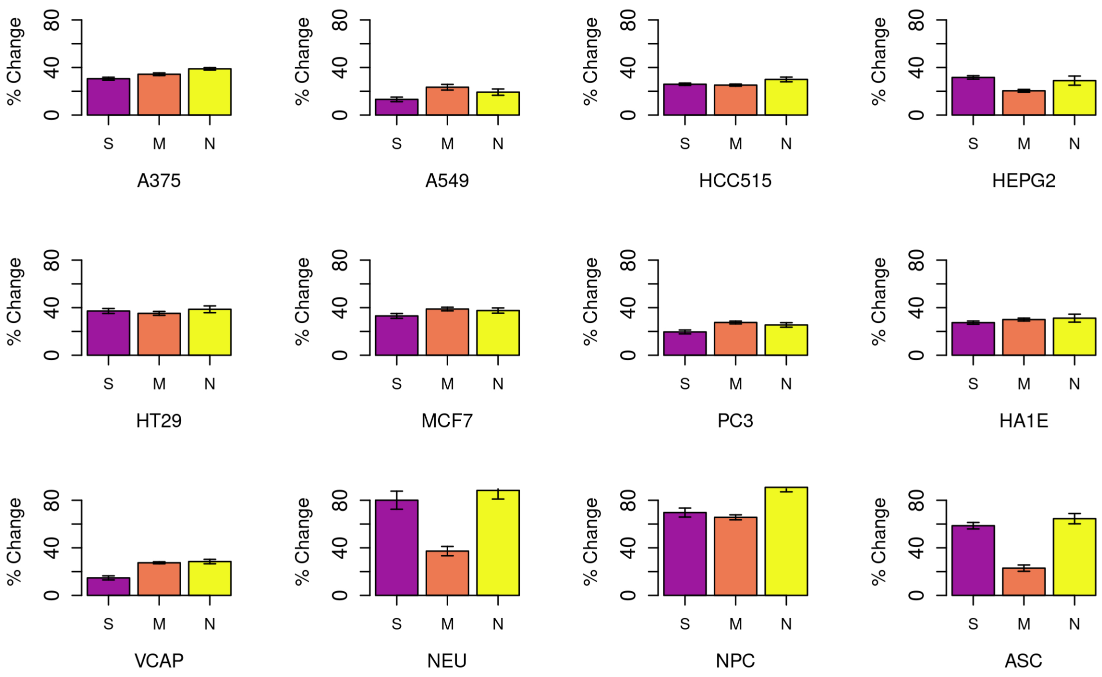

Figure S4: Percent change in Negative connectivity WSpe across all genes and drugs, for each of the 12 cell lines in the complete data set. Methods are denoted by single-letter labels: N : Neighborhood collaborative filtering; S : SVD; M : Median of medians; T : Tissue-agnostic (baseline method). Error bars show variation across cross validation runs.

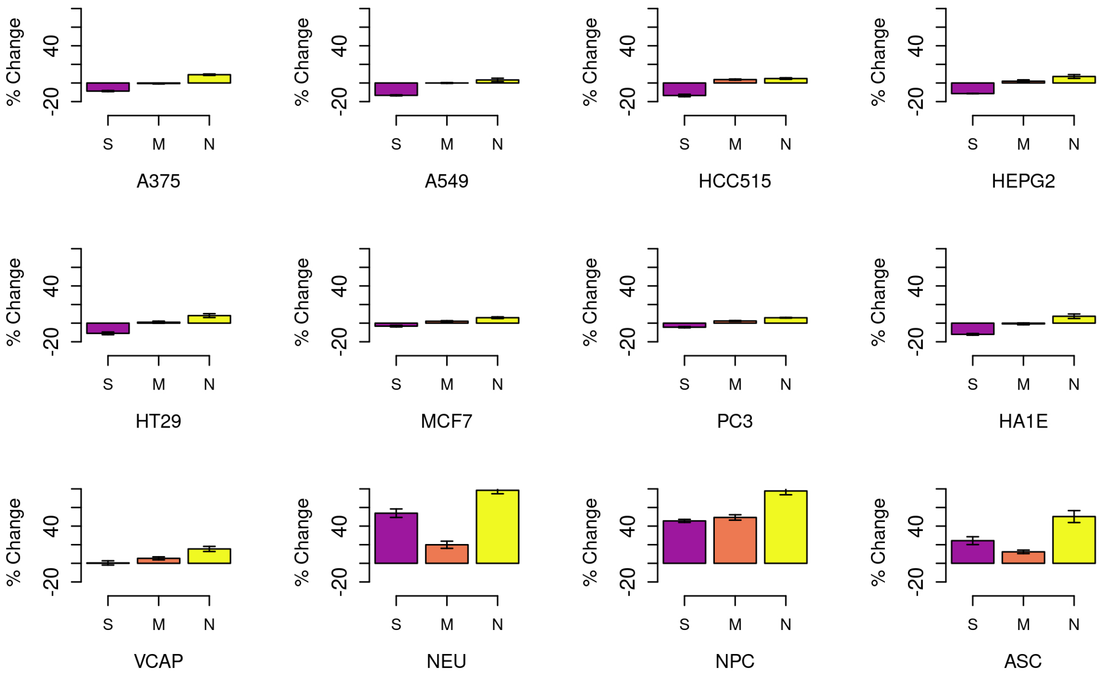

Figure S5: Percent change in Positive connectivity WSpe across all genes and drugs, for each of the 12 cell lines in the complete data set. Methods are denoted by single-letter labels: N : Neighborhood collaborative filtering; S : SVD; M : Median of medians; T : Tissue-agnostic (baseline method). Error bars show variation across cross validation runs.

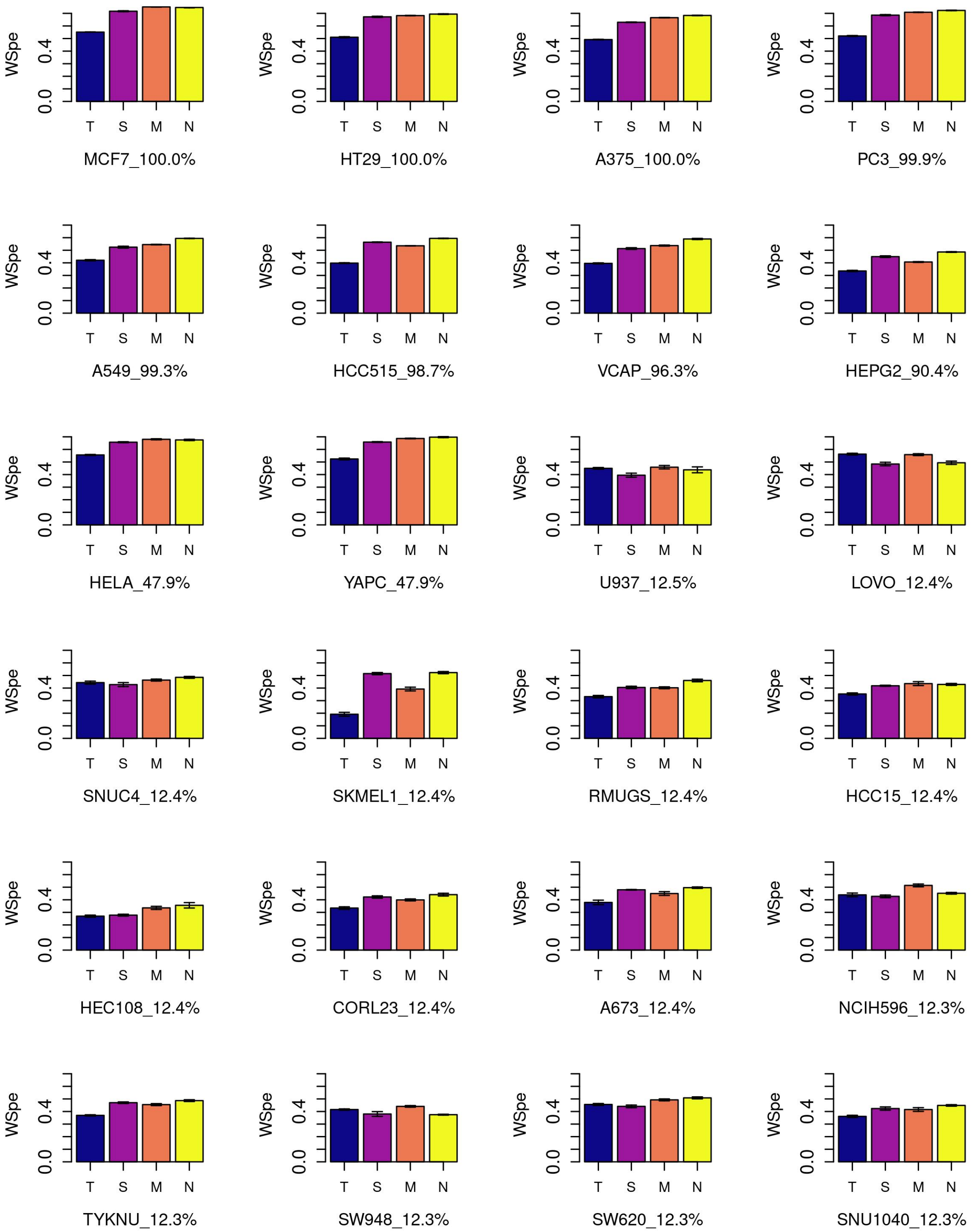
WSpe
0.0
0.4
T S M N
MCF7_100.0%
HT29_100.0%
A375_100.0%
PC3_99.9%
A549_99.3%
HCC515_98.7%
VCAP_96.3%
HEPG2_90.4%
HELA_47.9%
YAPC_47.9%
U937_12.5%
LOVO_12.4%
SNUC4_12.4%
SKMEL1_12.4%
RMUGS_12.4%
HCC15_12.4%
HEC108_12.4%
CORL23_12.4%
A673_12.4%
NCIH596_12.3%
TYKNU_12.3%
SW948_12.3%
SW620_12.3%
SNU1040_12.3%

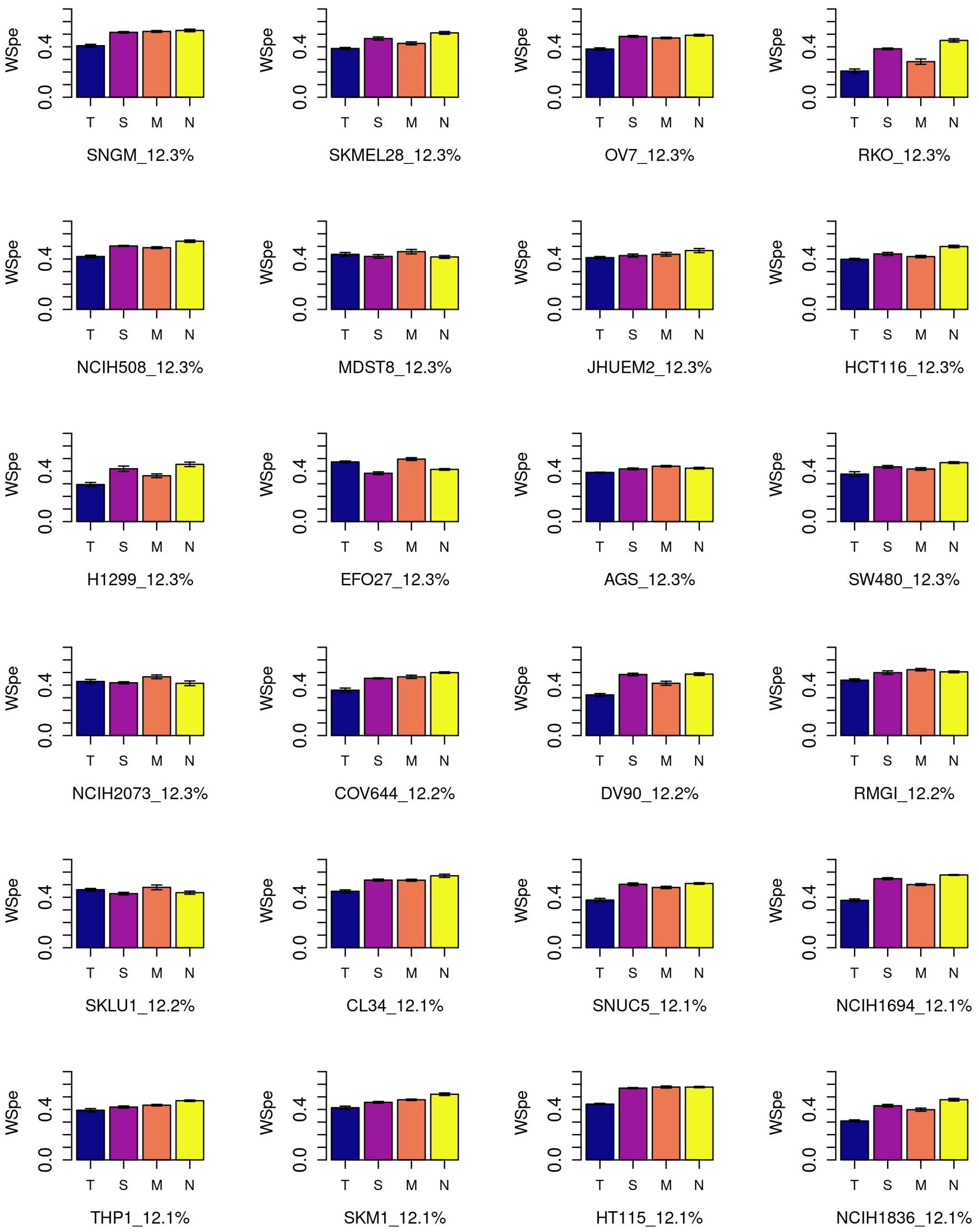
WSpe
0.0
0.4
T
S
M
N
SNGM_12.3%
SKMEL28_12.3%
OV7_12.3%
RKO_12.3%
NCIH508_12.3%
MDST8_12.3%
JHUEM2_12.3%
HCT116_12.3%
H1299_12.3%
EFO27_12.3%
AGS_12.3%
SW480_12.3%
NCIH2073_12.3%
COV644_12.2%
DV90_12.2%
RMGI_12.2%
SKLU1_12.2%
CL34_12.1%
SNUC5_12.1%
NCIH1694_12.1%
THP1_12.1%
SKM1_12.1%
HT115_12.1%
NCIH1836_12.1%

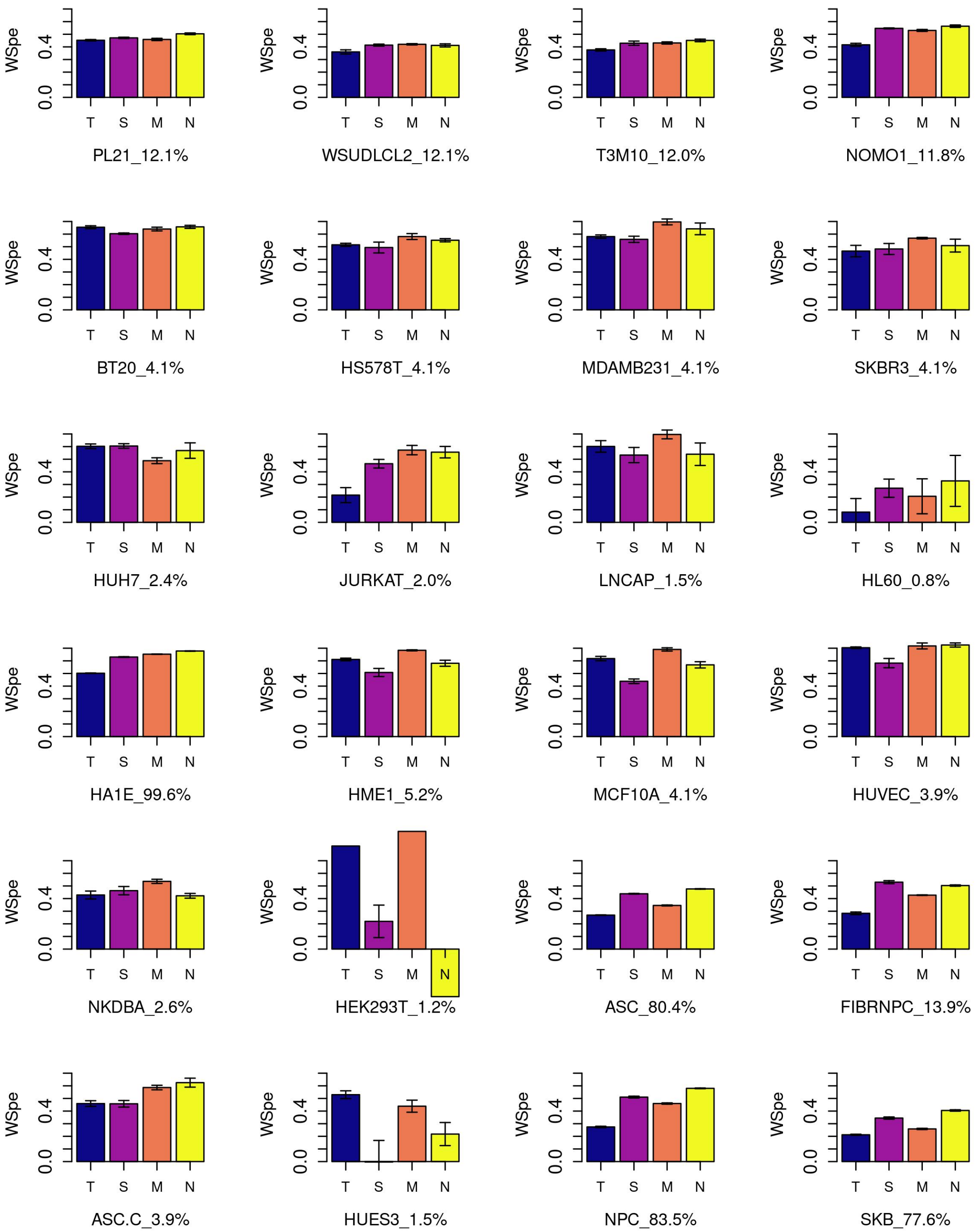
WSpe
0.0
0.4
T S M N
PL21_12.1%
WSUDLCL2_12.1%
T3M10_12.0%
NOMO1_11.8%
BT20_4.1%
HS578T_4.1%
MDAMB231_4.1%
SKBR3_4.1%
HUH7_2.4%
JURKAT_2.0%
LNCAP_1.5%
HL60_0.8%
HA1E_99.6%
HME1_5.2%
MCF10A_4.1%
HUVEC_3.9%
NKDBA_2.6%
HEK293T_1.2%
ASC_80.4%
FIBRNPC_13.9%
ASC.C_3.9%
HUES3_1.5%
NPC_83.5%
SKB_77.6%

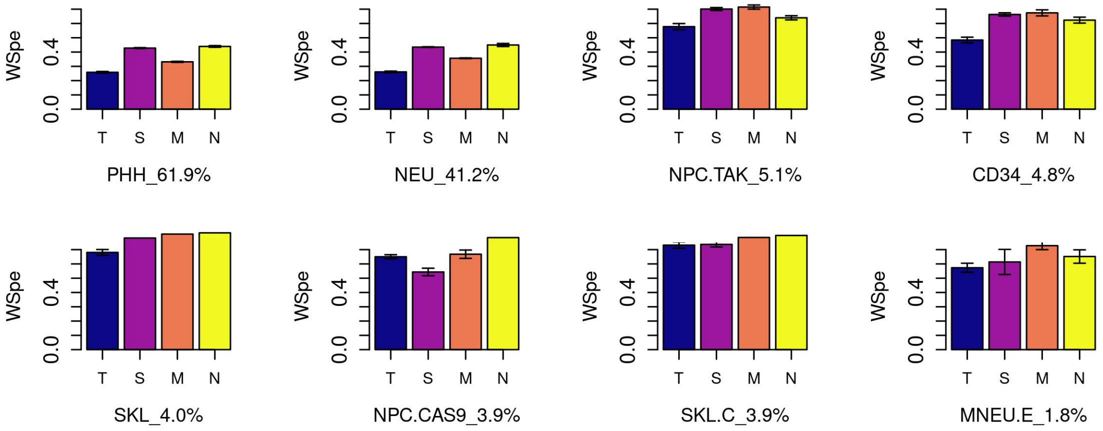

Figure S6: Negative Weighted Spearman connectivity correlation across all genes and drugs, for each of the 80 cell lines in the sparse matrix. Methods are denoted by single-letter labels: N : neighborhood collaborative filtering; S : SVD; M : Median of medians; T : Tissue-agnostic. Ordered by cell type (cancer, immortalized, stem and primary) and then by percentage of drugs profiled in each cell. Error bars show variation across cross validation runs.

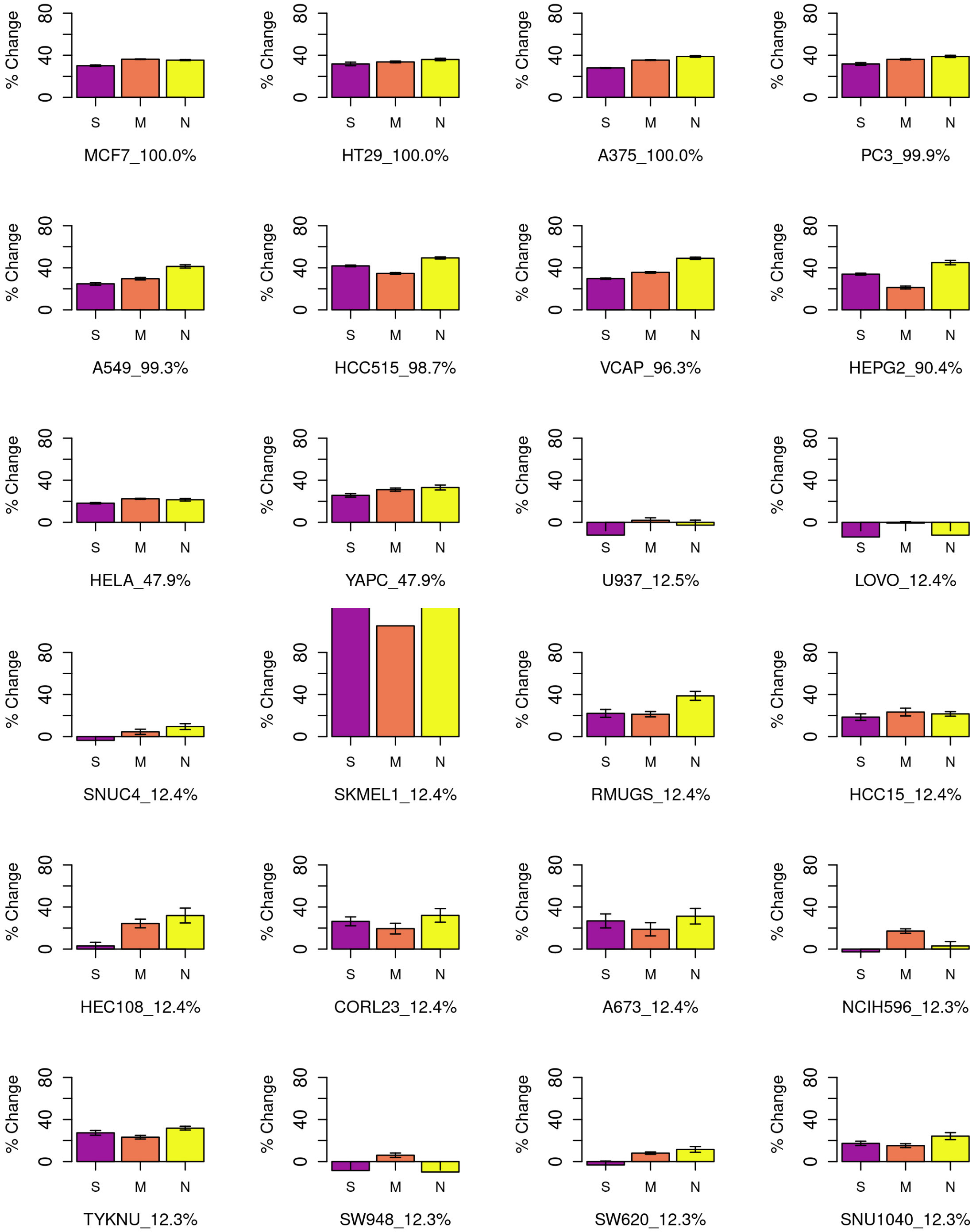
% Change
0
40
80
S
M
N
MCF7_100.0%
HT29_100.0%
A375_100.0%
PC3_99.9%
A549_99.3%
HCC515_98.7%
VCAP_96.3%
HEPG2_90.4%
HELA_47.9%
YAPC_47.9%
U937_12.5%
LOVO_12.4%
SNUC4_12.4%
SKMEL1_12.4%
RMUGS_12.4%
HCC15_12.4%
HEC108_12.4%
CORL23_12.4%
A673_12.4%
NCIH596_12.3%
TYKNU_12.3%
SW948_12.3%
SW620_12.3%
SNU1040_12.3%

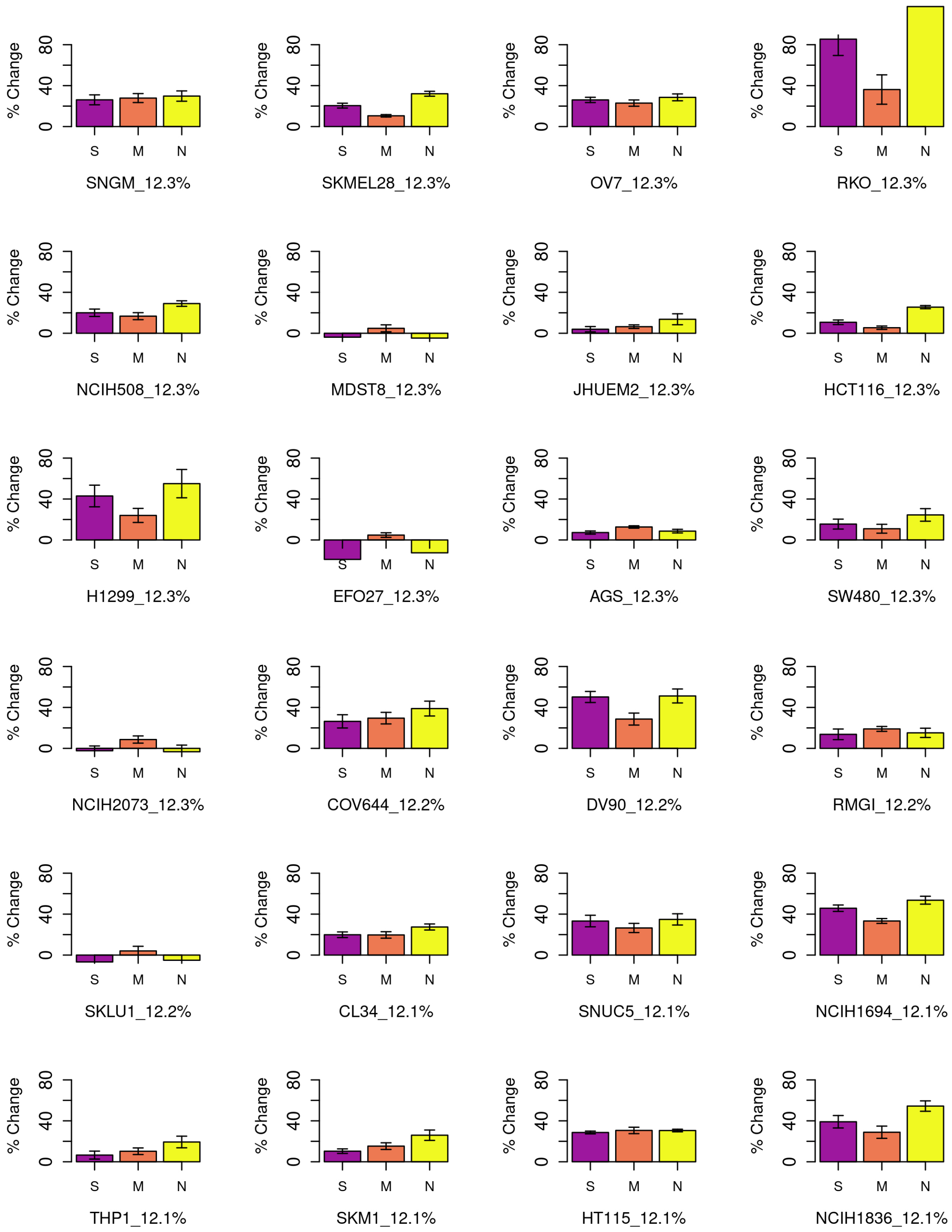
% Change
0
40
80
S
M
N
SNGM_12.3%
SKMEL28_12.3%
OV7_12.3%
RKO_12.3%
NCIH508_12.3%
MDST8_12.3%
JHUEM2_12.3%
HCT116_12.3%
H1299_12.3%
EFO27_12.3%
AGS_12.3%
SW480_12.3%
NCIH2073_12.3%
COV644_12.2%
DV90_12.2%
RMGI_12.2%
SKLU1_12.2%
CL34_12.1%
SNUC5_12.1%
NCIH1694_12.1%
THP1_12.1%
SKM1_12.1%
HT115_12.1%
NCIH1836_12.1%

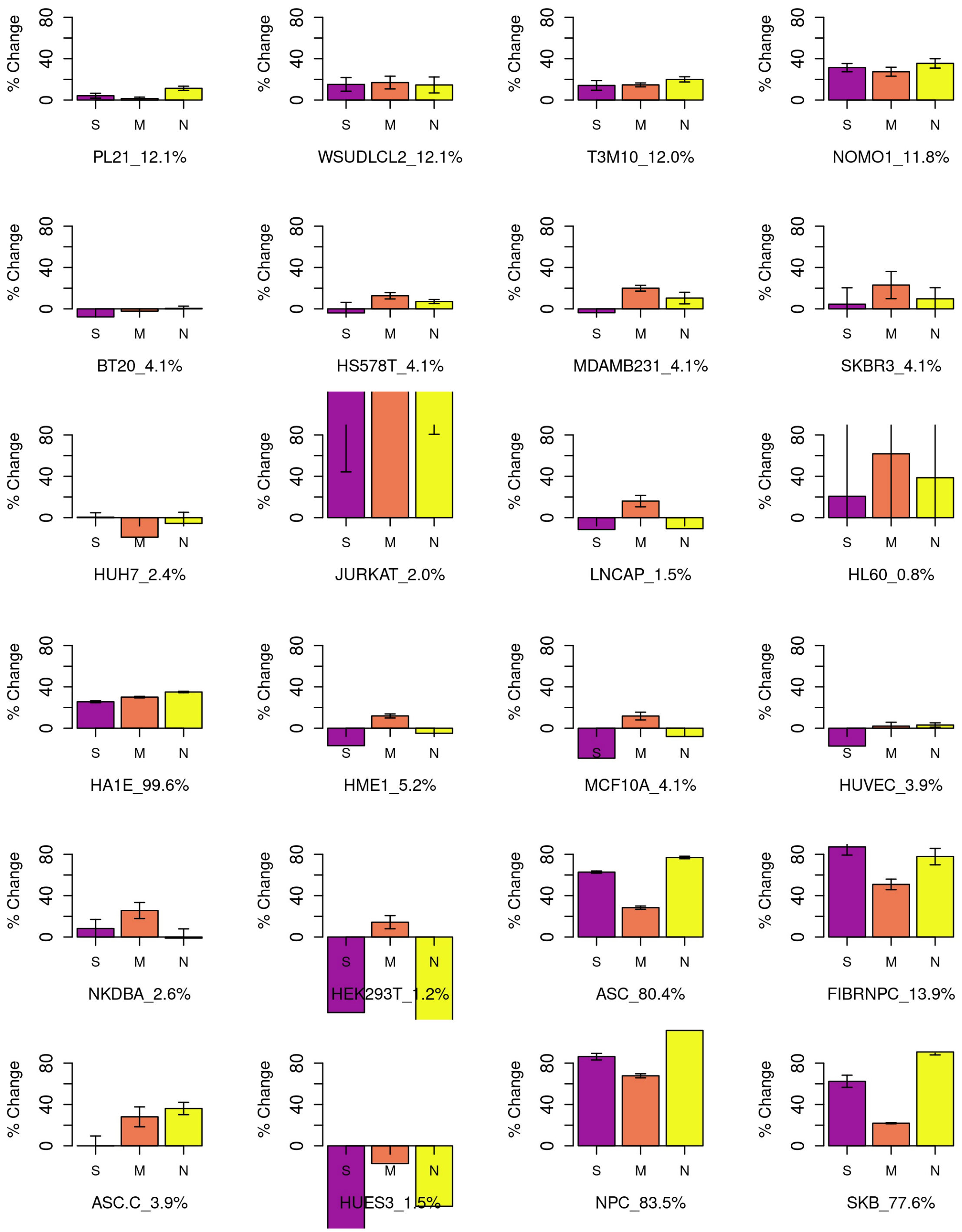
% Change
0
40
80
S
M
N
PL21_12.1%
WSUDLCL2_12.1%
T3M10_12.0%
NOMO1_11.8%
BT20_4.1%
HS578T_4.1%
MDAMB231_4.1%
SKBR3_4.1%
HUH7_2.4%
JURKAT_2.0%
LNCAP_1.5%
HL60_0.8%
HA1E_99.6%
HME1_5.2%
MCF10A_4.1%
HUVEC_3.9%
NKDBA_2.6%
HEK293T_1.2%
ASC_80.4%
FIBRNPC_13.9%
ASC.C_3.9%
HUES3_1.5%
NPC_83.5%
SKB_77.6%

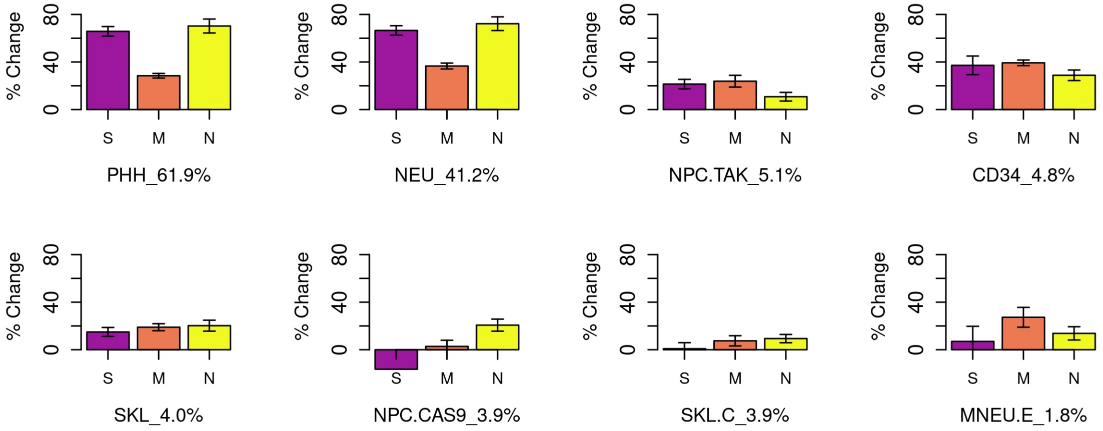

Figure S7: Percent change in negative Weighted Spearman connectivity correlation compared to the tissue-agnostic method. Methods are denoted by single-letter labels: N : neighborhood collaborative filtering; S : SVD; M : Median of medians; T : Tissue-agnostic. Ordered by cell type (cancer, immortalized, stem and primary) and then by percentage of drugs profiled in each cell. Error bars show variation across cross validation runs.

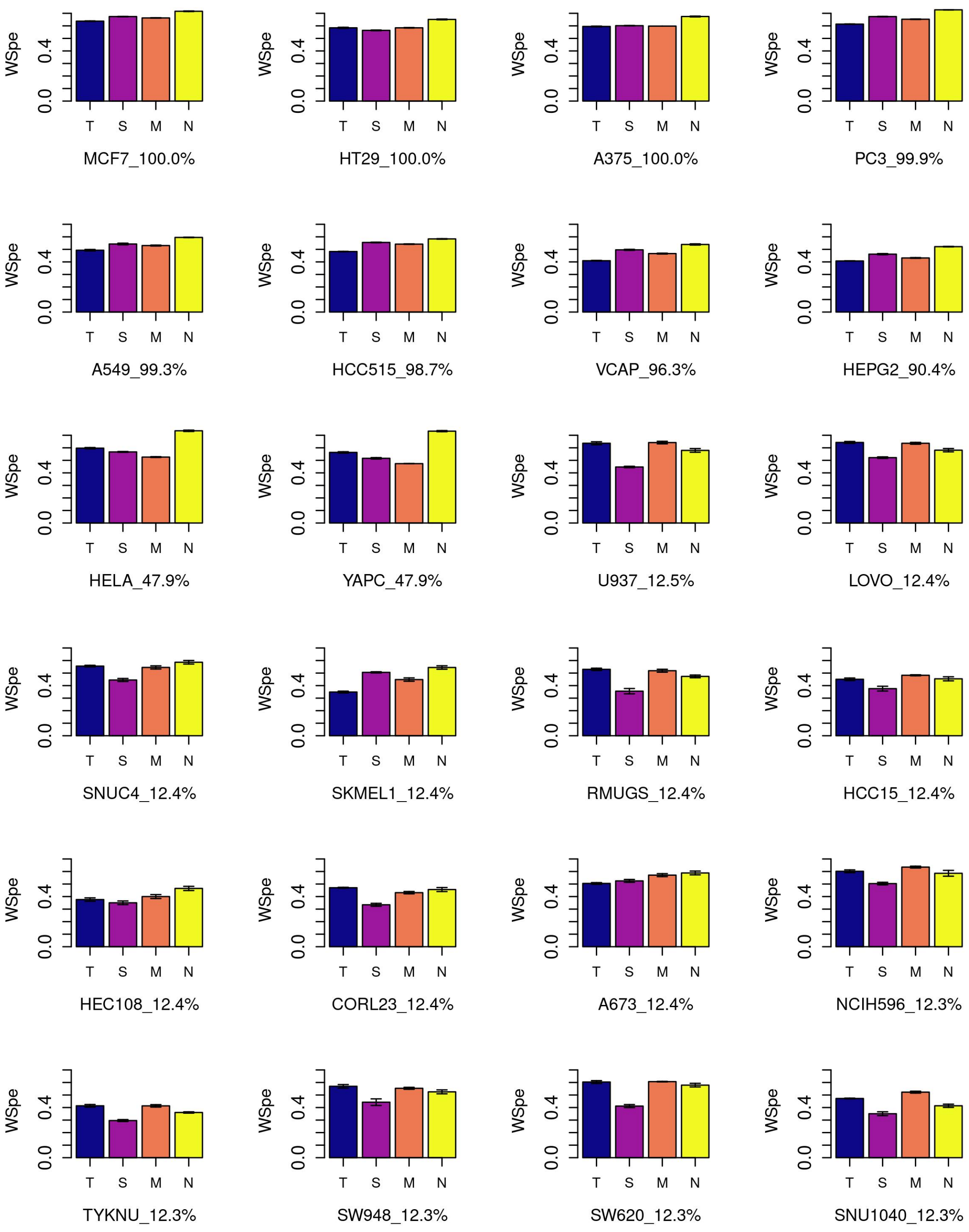
WSpe
0.0
0.4
T S M N
MCF7_100.0%
HT29_100.0%
A375_100.0%
PC3_99.9%
A549_99.3%
HCC515_98.7%
VCAP_96.3%
HEPG2_90.4%
HELA_47.9%
YAPC_47.9%
U937_12.5%
LOVO_12.4%
SNUC4_12.4%
SKMEL1_12.4%
RMUGS_12.4%
HCC15_12.4%
HEC108_12.4%
CORL23_12.4%
A673_12.4%
NCIH596_12.3%
TYKNU_12.3%
SW948_12.3%
SW620_12.3%
SNU1040_12.3%

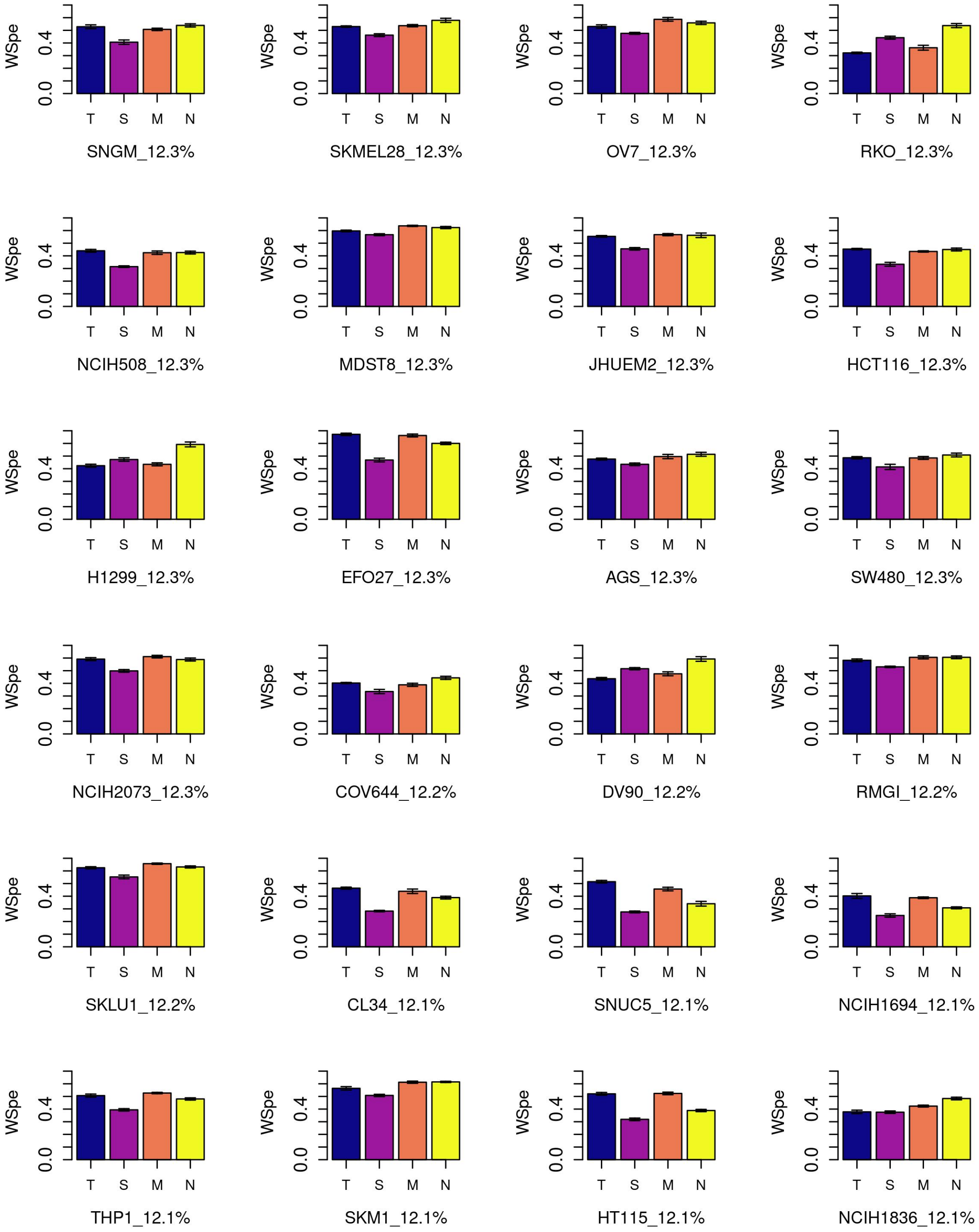
WSpe
0.0
0.4
T
S
M
N
SNGM_12.3%
SKMEL28_12.3%
OV7_12.3%
RKO_12.3%
NCIH508_12.3%
MDST8_12.3%
JHUEM2_12.3%
HCT116_12.3%
H1299_12.3%
EFO27_12.3%
AGS_12.3%
SW480_12.3%
NCIH2073_12.3%
COV644_12.2%
DV90_12.2%
RMGI_12.2%
SKLU1_12.2%
CL34_12.1%
SNUC5_12.1%
NCIH1694_12.1%
THP1_12.1%
SKM1_12.1%
HT115_12.1%
NCIH1836_12.1%

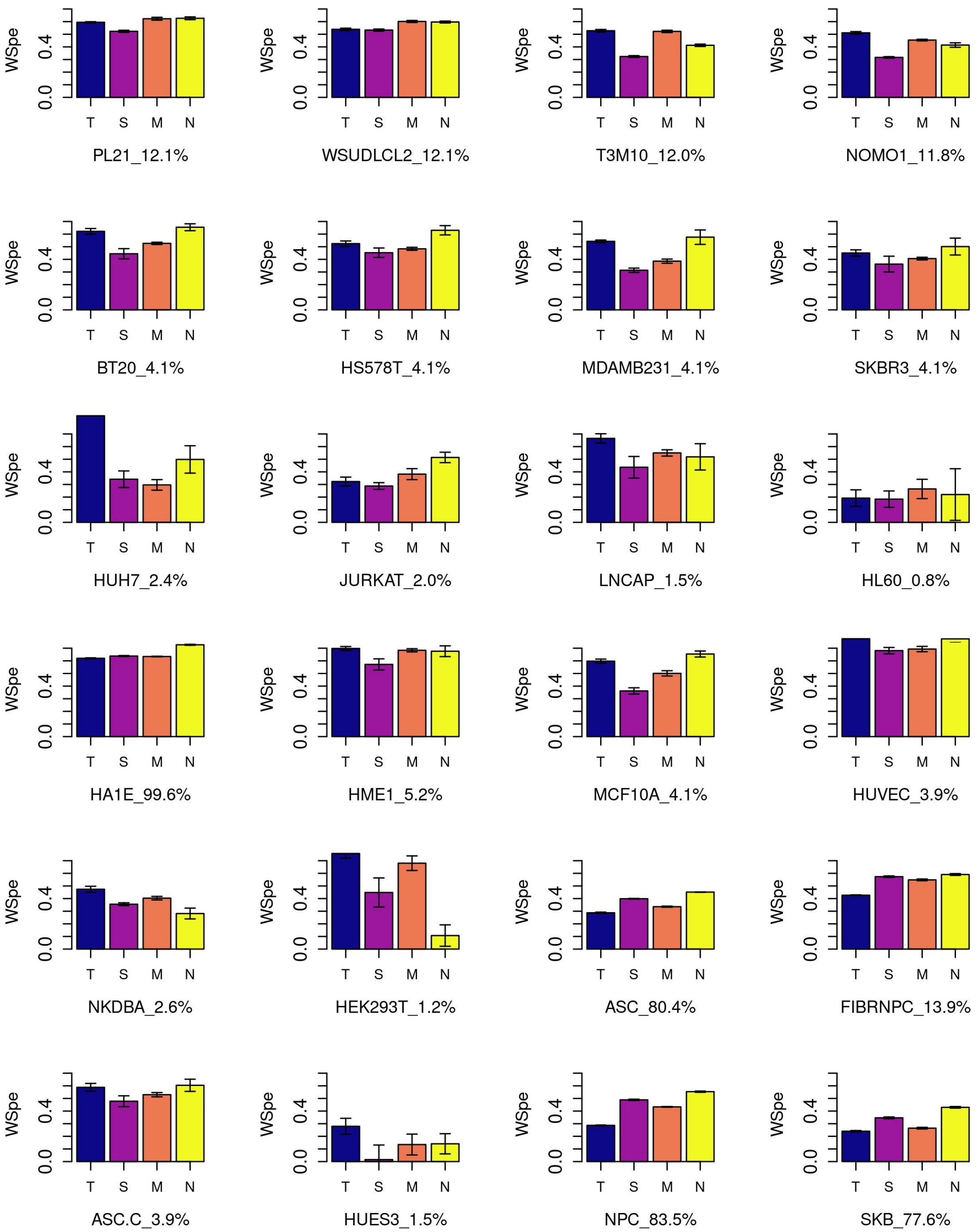
WSpe
0.0
0.4
T S M N
PL21_12.1%
WSUDLCL2_12.1%
T3M10_12.0%
NOMO1_11.8%
BT20_4.1%
HS578T_4.1%
MDAMB231_4.1%
SKBR3_4.1%
HUH7_2.4%
JURKAT_2.0%
LNCAP_1.5%
HL60_0.8%
HA1E_99.6%
HME1_5.2%
MCF10A_4.1%
HUVEC_3.9%
NKDBA_2.6%
HEK293T_1.2%
ASC_80.4%
FIBRNPC_13.9%
ASC.C_3.9%
HUES3_1.5%
NPC_83.5%
SKB_77.6%

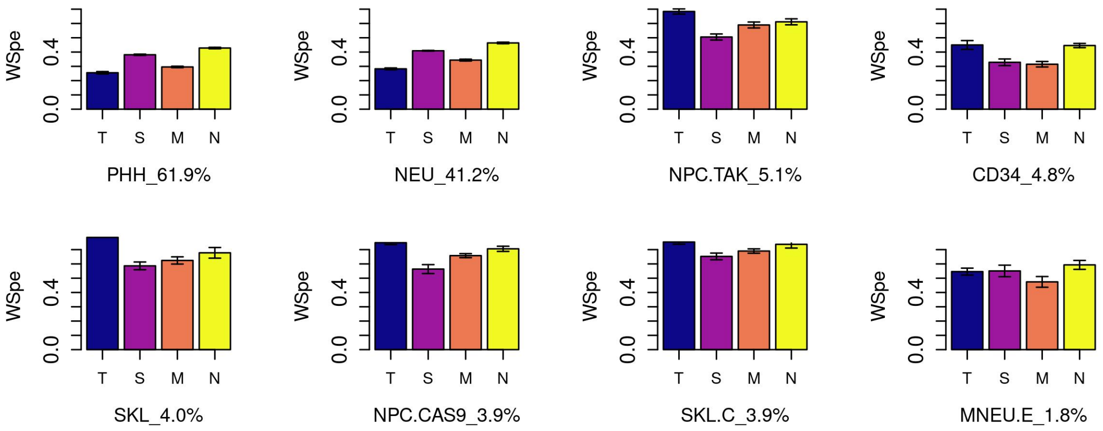

Figure S8: Positive Weighted Spearman connectivity correlation across all genes and drugs, for each of the 80 cell lines in the sparse matrix. Methods are denoted by single-letter labels: N : neighborhood collaborative filtering; S : SVD; M : Median of medians; T : Tissue-agnostic. Ordered by cell type (cancer, immortalized, stem and primary) and then by percentage of drugs profiled in each cell. Error bars show variation across cross validation runs.

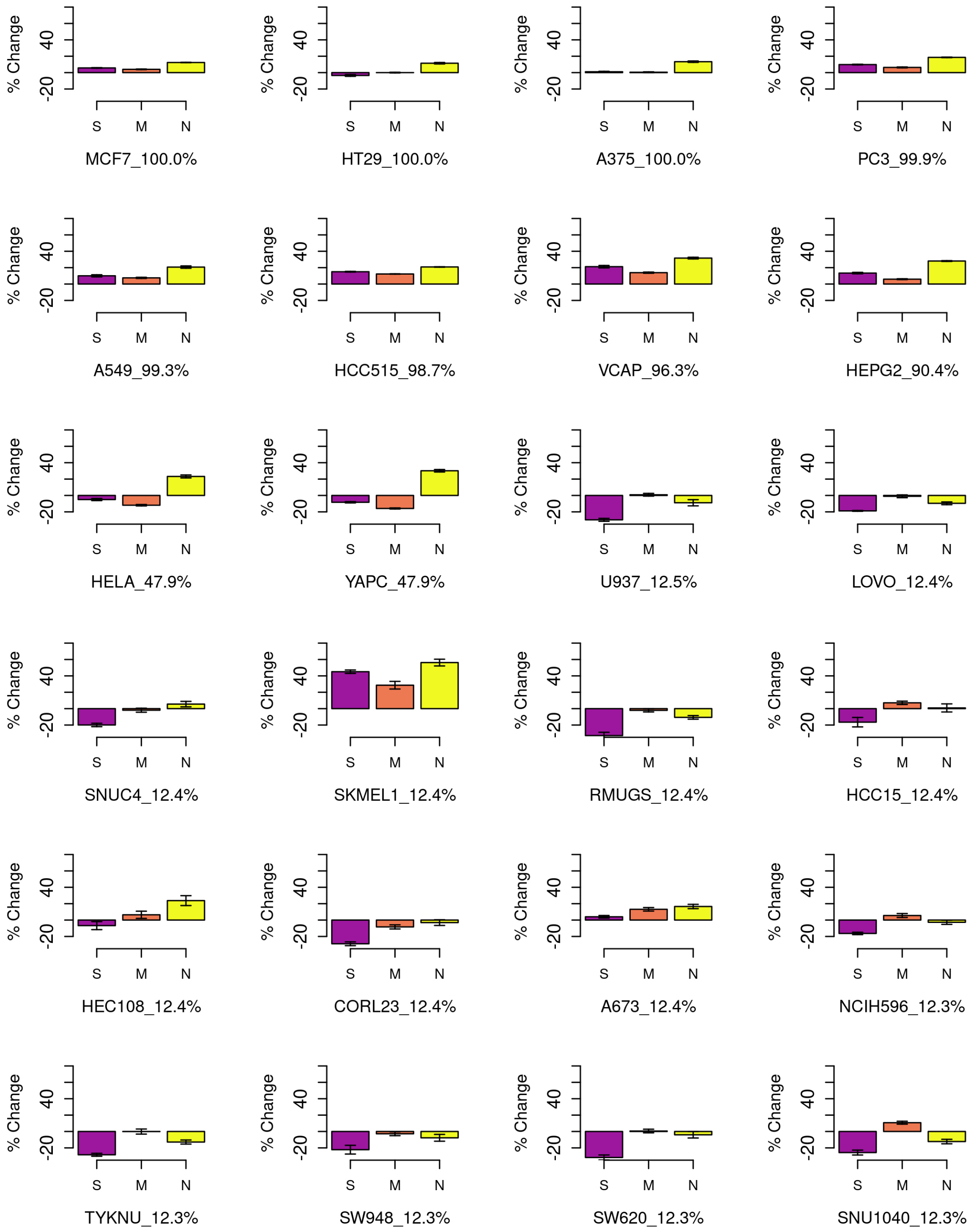
% Change
40
-20
S
M
N
MCF7_100.0%
HT29_100.0%
A375_100.0%
PC3_99.9%
A549_99.3%
HCC515_98.7%
VCAP_96.3%
HEPG2_90.4%
HELA_47.9%
YAPC_47.9%
U937_12.5%
LOVO_12.4%
SNUC4_12.4%
SKMEL1_12.4%
RMUGS_12.4%
HCC15_12.4%
HEC108_12.4%
CORL23_12.4%
A673_12.4%
NCIH596_12.3%
TYKNU_12.3%
SW948_12.3%
SW620_12.3%
SNU1040_12.3%

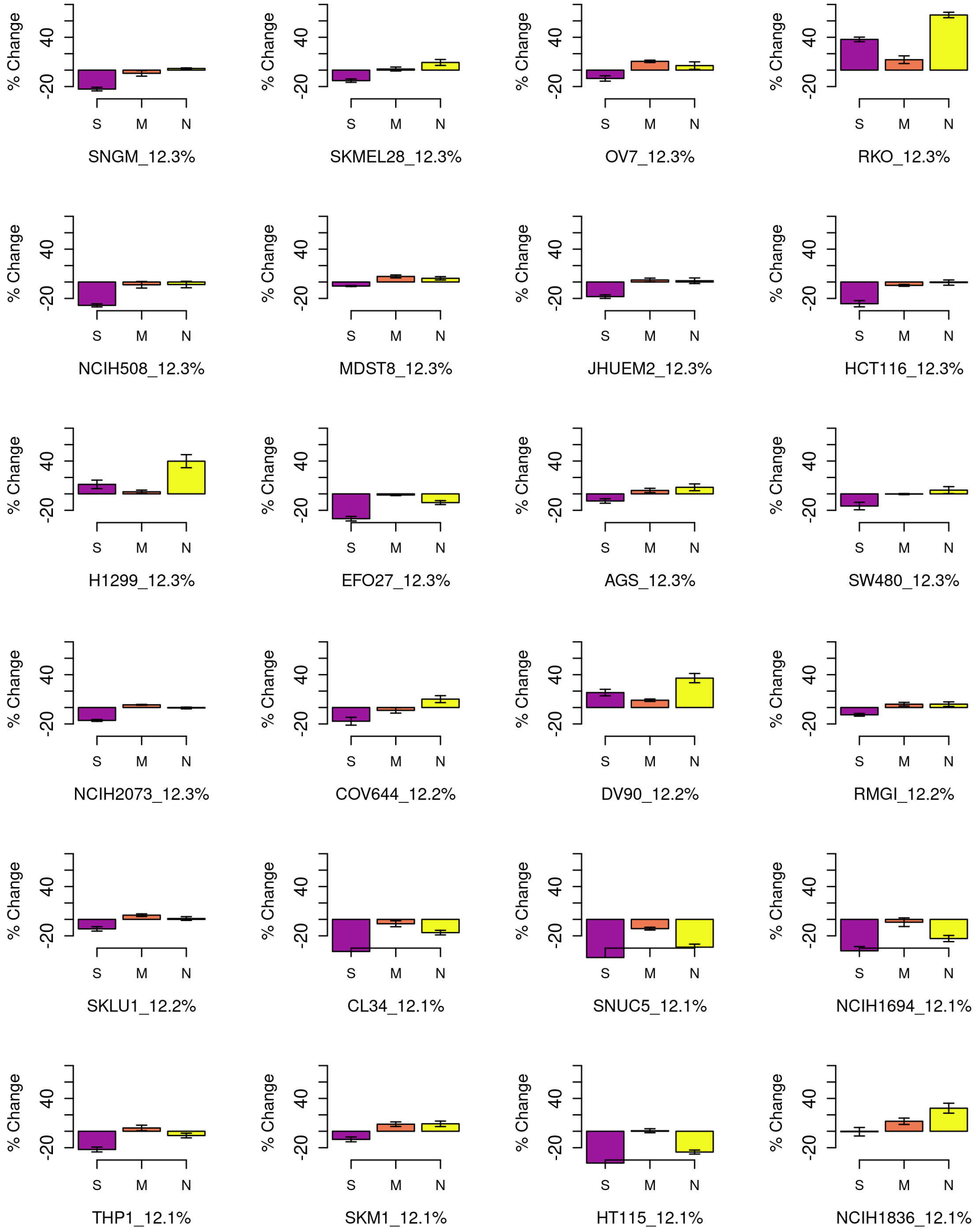
% Change
40
-20
S
M
N
SNGM_12.3%
SKMEL28_12.3%
OV7_12.3%
RKO_12.3%
NCIH508_12.3%
MDST8_12.3%
JHUEM2_12.3%
HCT116_12.3%
H1299_12.3%
EFO27_12.3%
AGS_12.3%
SW480_12.3%
NCIH2073_12.3%
COV644_12.2%
DV90_12.2%
RMGI_12.2%
SKLU1_12.2%
CL34_12.1%
SNUC5_12.1%
NCIH1694_12.1%
THP1_12.1%
SKM1_12.1%
HT115_12.1%
NCIH1836_12.1%

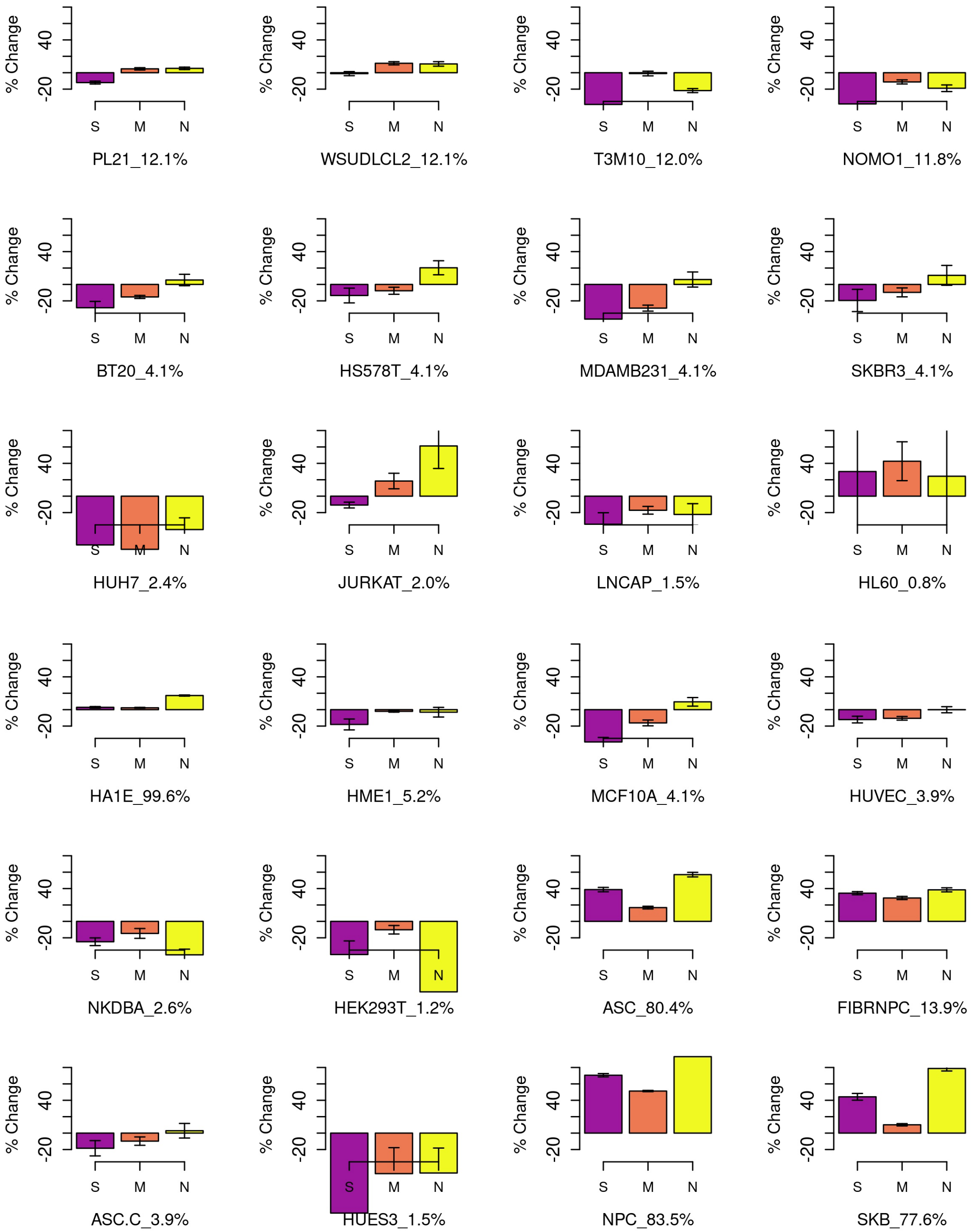
% Change
40
-20
S
M
N
PL21_12.1%
WSUDLCL2_12.1%
T3M10_12.0%
NOMO1_11.8%
BT20_4.1%
HS578T_4.1%
MDAMB231_4.1%
SKBR3_4.1%
HUH7_2.4%
JURKAT_2.0%
LNCAP_1.5%
HL60_0.8%
HA1E_99.6%
HME1_5.2%
MCF10A_4.1%
HUVEC_3.9%
NKDBA_2.6%
HEK293T_1.2%
ASC_80.4%
FIBRNPC_13.9%
ASC.C_3.9%
HUES3_1.5%
NPC_83.5%
SKB_77.6%

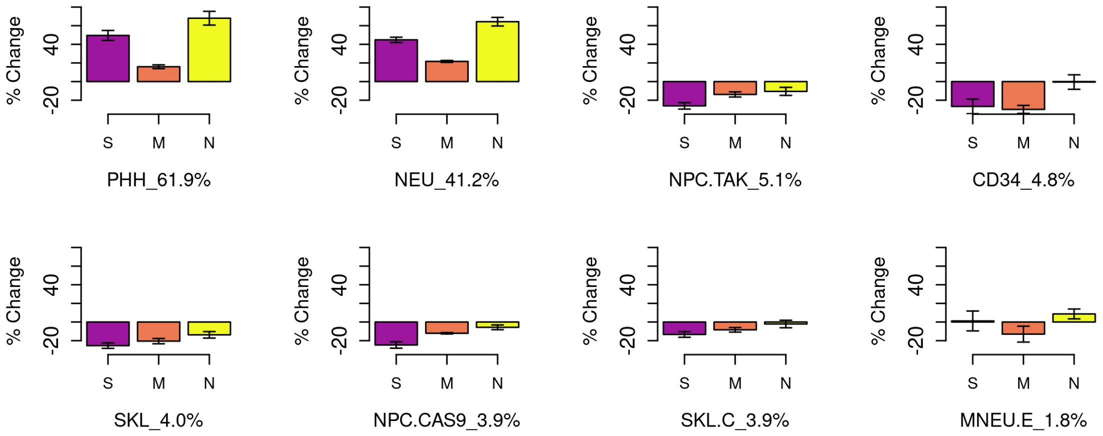

Figure S9: Percent change in positive Weighted Spearman connectivity correlation compared to the tissue-agnostic method. Methods are denoted by single-letter labels: N : neighborhood collaborative filtering; S : SVD; M : Median of medians; T : Tissue-agnostic. Ordered by cell type (cancer, immortalized, stem and primary) and then by percentage of drugs profiled in each cell. Error bars show variation across cross validation runs.

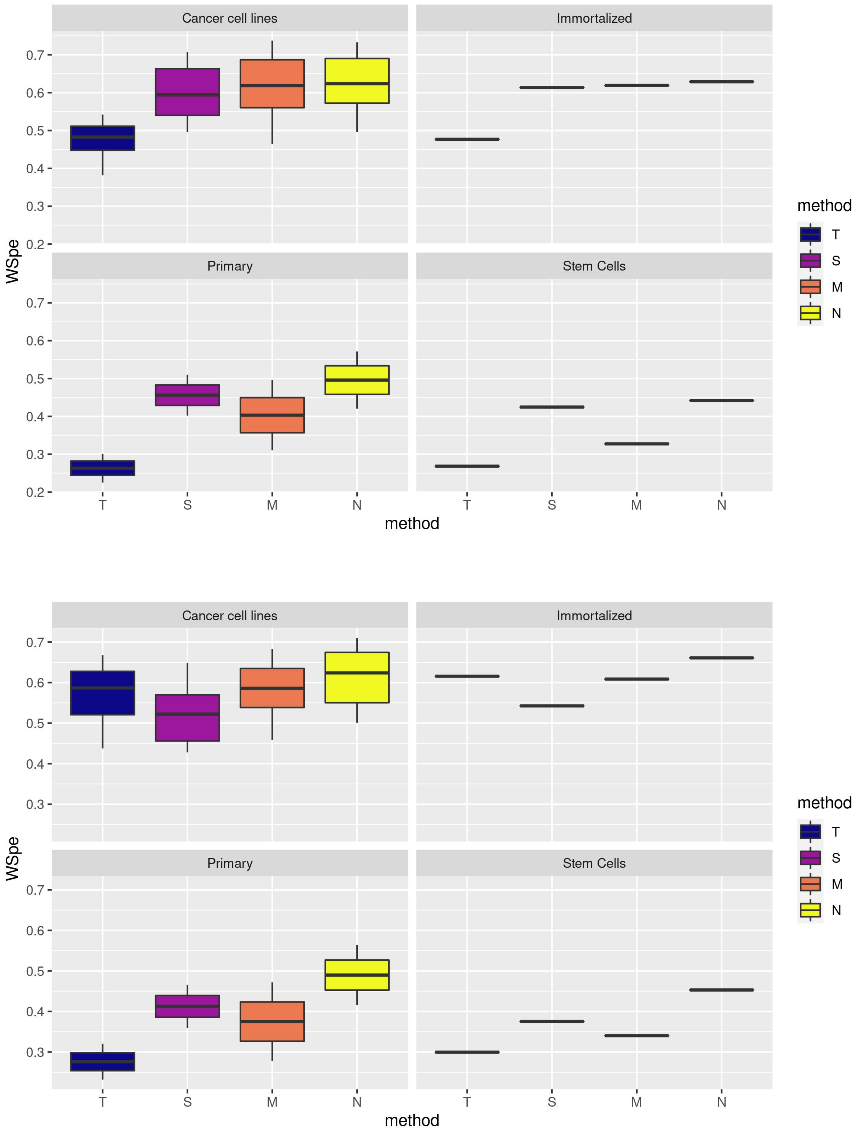

Figure S10: By cell type, boxplots of median WSpe distributions in each cell, for negative (top) and positive (bottom) connectivity, across all drugs in the complete data set. Horizontal lines instead of boxes indicate that there is only one cell of that type in the data set.

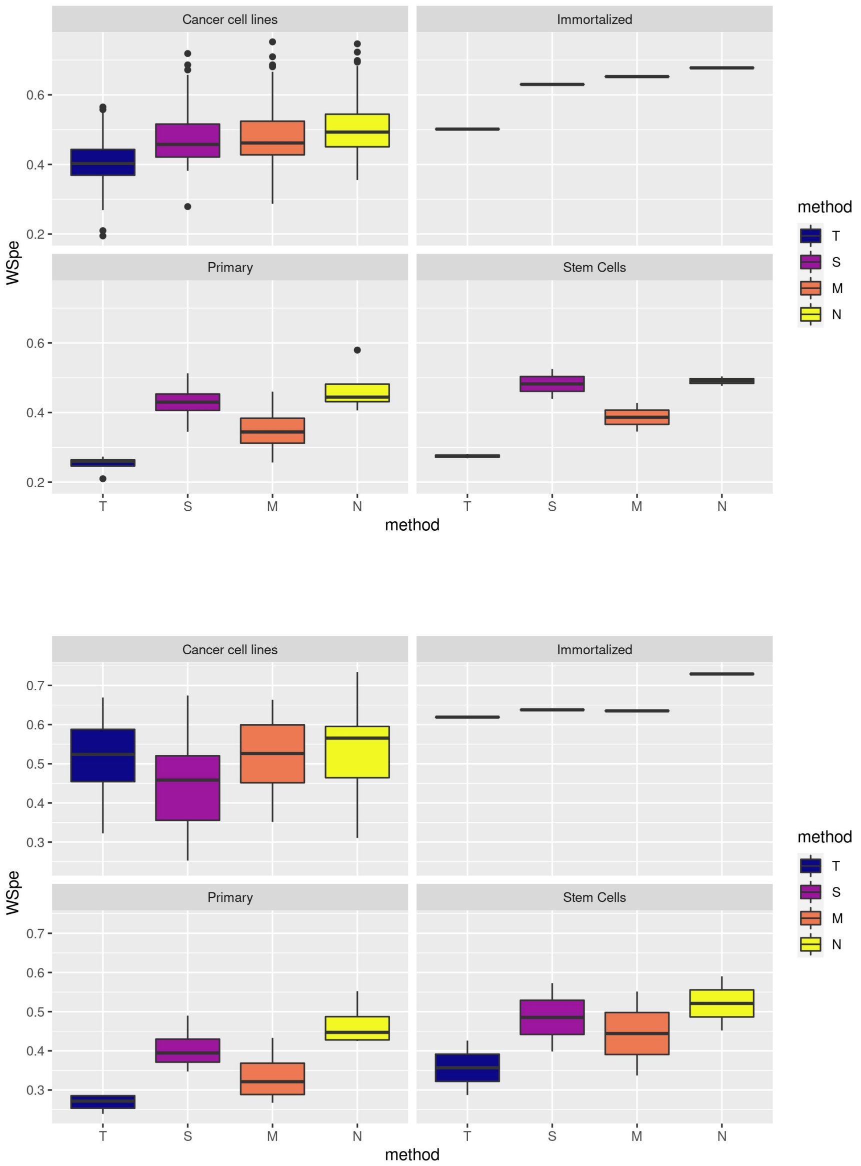

Figure S11: By cell type, boxplots of median WSpe distributions in each cell, for negative (top) and positive (bottom) connectivity, across all drugs in the sparse data set. Because of the unreliability of the cell-specific imputation methods when fewer than 10% of drugs are present, only cells with at least 10% of the compounds assayed are included here. Horizontal lines indicate that there is only one cell of that type in the data set.

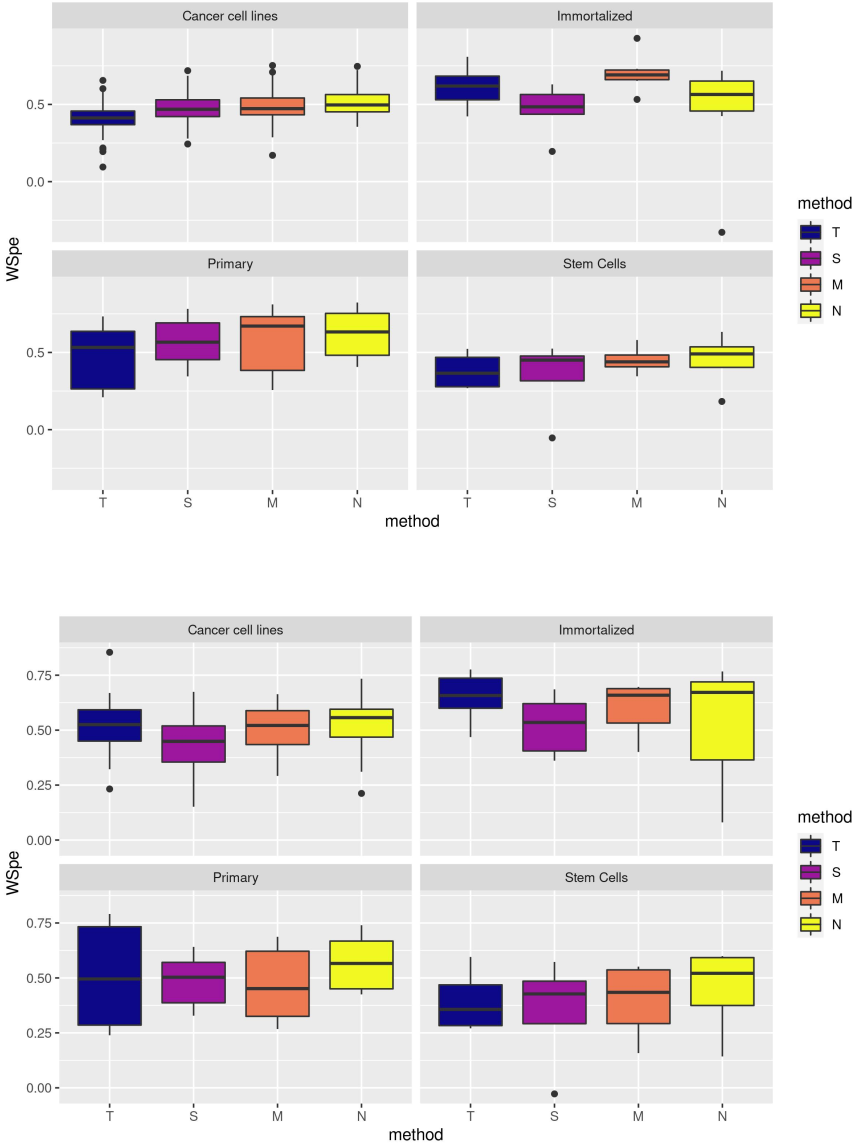

Figure S12: By cell type, boxplots of WSpe distributions for negative (top) and positive (bottom) connectivity, averaged across all replicates and drugs. These are noisier than in the previous figure because *all* cells, including those with data for under 10% of the drugs, are included. Note that these noisy cases with too little data account for half of the stem cells, 3/5 of the primary cells, and 5/6 of the immortalized non-cancer cells, explaining the increased variability compared to the prior boxplot.

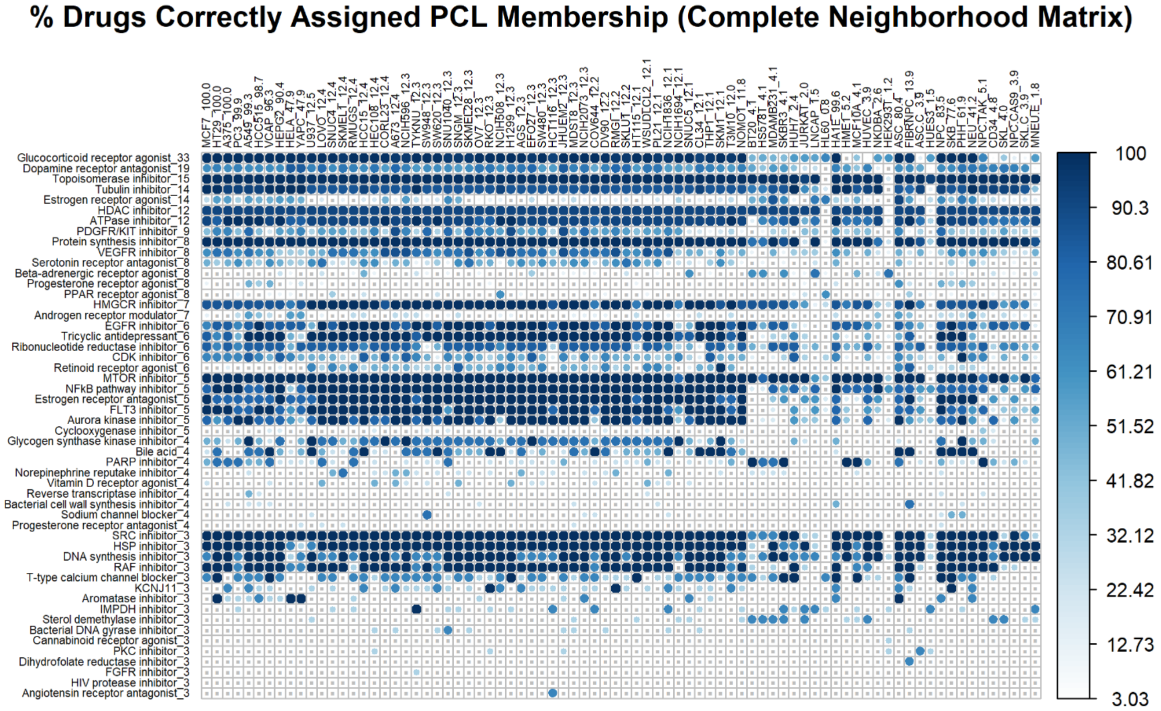

Figure S13: Percent of drugs correctly expressing strong connectivity to their drug class using the fully imputed sparse matrix by the neighborhood approach. Cells are organized by cell type (cancer, immortalized, stem and primary) and ordered by percentage of drugs profiled in each cell. PCL sets are ordered by the number of drugs in each set that are in the sparse matrix. The darker the shade of blue, the higher percentage of drugs with statistically significant NCS scores. Grey dots represent PCL/cell combinations in which there were no statistically significant NCS scores. In the main text, primary cells were pulled out and the plot was transposed for readability.

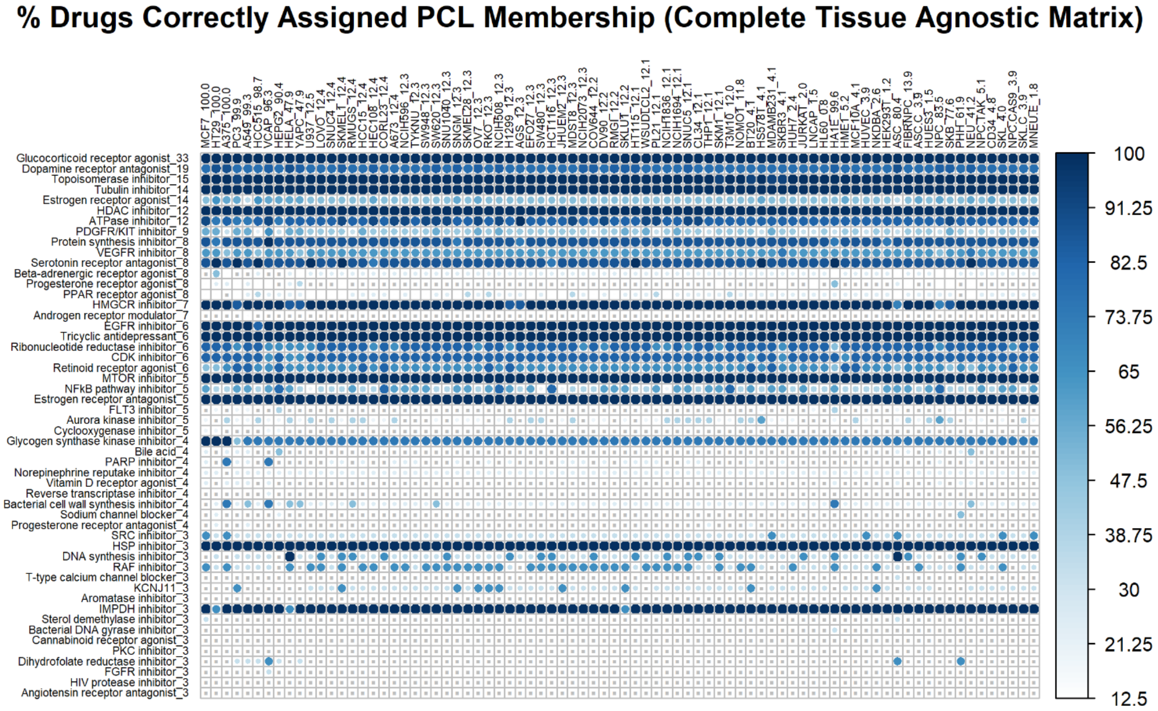

Figure S14: Percent of drugs correctly expressing strong connectivity to their drug class using the fully imputed sparse matrix by the tissue agnostic approach. Cells are organized by cell type (cancer, immortalized, stem and primary) and ordered by percentage of drugs profiled in each cell. PCL sets are ordered by the number of drugs in each set that are in the sparse matrix. The darker the shade of blue, the higher percentage of drugs with statistically significant NCS scores. Grey dots represent PCL/cell combinations in which there were no statistically significant NCS scores. In the main text, primary cells were pulled out and the plot was transposed for readability.
